# A fuzzy logic decision support model for climate-driven biomass loss risk in western Oregon and Washington

**DOI:** 10.1371/journal.pone.0222051

**Published:** 2019-10-25

**Authors:** T. Sheehan, D. Bachelet

**Affiliations:** 1 Conservation Biology Institute, Corvallis, Oregon, United States of America; 2 Environmental Sciences Program, Oregon State University, Corvallis, Oregon, United States of America; 3 Department of Biological and Ecological Engineering, Oregon State University, Corvallis, Oregon, United States of America; Peking University, CHINA

## Abstract

Dynamic global vegetation model (DGVM) projections are often put forth to aid resource managers in climate change-related decision making. However, interpreting model results and understanding their uncertainty can be difficult. Sources of uncertainty include embedded assumptions about atmospheric CO_2_ levels, uncertain climate projections driving DGVMs, and DGVM algorithm selection. For western Oregon and Washington, we implemented an Environmental Evaluation Modeling System (EEMS) decision support model using MC2 DGVM results to characterize biomass loss risk. MC2 results were driven by climate projections from 20 General Circulation Models (GCMs) and Earth System Models (ESMs), under Representative Concentration Pathways (RCPs) 4.5 and 8.5, with and without assumed fire suppression, for three different time periods. We produced maps of mean, minimum, and maximum biomass loss risk and uncertainty for each RCP / +/- fire suppression / time period. We characterized the uncertainty due to RCP, fire suppression, and climate projection choice. Finally, we evaluated whether fire or climate maladaptation mortality was the dominant driver of risk for each model run. The risk of biomass loss generally increases in current high biomass areas within the study region through time. The pattern of increased risk is generally south to north and upslope into the Coast and Cascade mountain ranges and along the coast. Uncertainty from climate future choice is greater than that attributable to RCP or +/- fire suppression. Fire dominates as the driving factor for biomass loss risk in more model runs than mortality. This method of interpreting DGVM results and the associated uncertainty provides managers with data in a form directly applicable to their concerns and should prove helpful in adaptive management planning.

## Introduction

Anthropogenic emissions have caused oceanic and atmospheric warming, diminished snow and ice, and rising sea level [1]. The effects of climate change vary regionally [1] and have already affected crop yields [2–5]), biodiversity [6–7], and wildfire risk [8–10]. In the Pacific Northwest of the conterminous United States (PNW), anthropogenic influences are the leading contributor to observed warming [11–12], with impacts including lower winter snowpack and increased wildfire risk [11]. Expected future warming in the PNW is projected to cause continued snowpack loss, increased risk of insect infestations [13], increased risk of wildfires, and changes in vegetation [11, 14].

Numerous studies within or including the PNW have projected climate-driven changes in vegetation, fire regime, pests, and forest productivity [14–25]. These studies have used a variety of methods and models, including Dynamic Global Vegetation Models (DGVMs) [14, 20, 25], statistical models [15–19], reconstruction of relationships between past climate, fire, and vegetation [23], observation and imputation [24], hybrid process and statistical models [21], and hybrid state and transition models [22]. While these studies present both spatial and regional model results, and in many cases, uncertainty associated with those results, the implications for higher level management decisions require interpretation.

Climate impacts have been a steadily growing research topic for over thirty years, and the focus on climate adaptation has seen a marked increase over the last decade [26] Uncertainty in future climate includes the unknown trend of CO_2_ concentrations, which in turn depend on political and economic decisions, and the wide range of future projections from GCMs and ESMs [1, 27–28]. The uncertainty in vegetation modeling results is due to the range of climate futures driving them [27–28], soil representation [29], parameter values based on 20^th^ century records [27–28], and model choice [28].

A common solution for resource managers faced with uncertainty is adaptive management [30–31], the “flexible decision making that can be adjusted in the face of uncertainties as outcomes from management actions and other events become better understood ([32] in [30]).” Accounting for and characterizing uncertainty are important aspects of adaptive management [28, 30–31].

**Fig 1:**
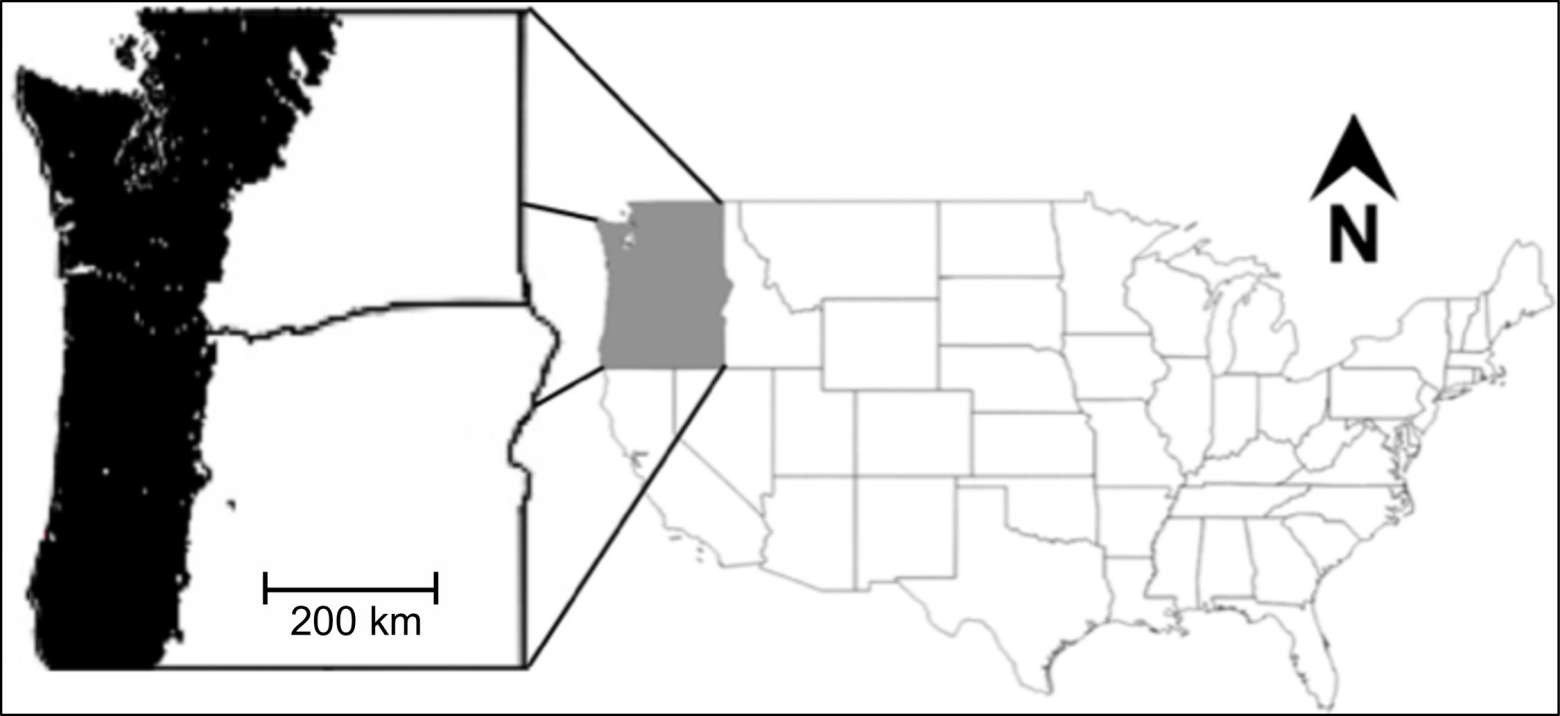
Study area. Portions of Oregon and Washington west of the Cascade Mountain Range crest.

In this study we report on a fuzzy logic model for assessing the risk of biomass loss due to climate change in western Oregon and Washington (Fig 1). We created a decision support model (DSM) to evaluate the risk of losing biomass under climate change projections. In the DSM we included results from 80 runs of the MC2 Dynamic Global Vegetation Model (DGVM) [33] as well as carbon stocks from [34]. We characterized uncertainty due to the diverse climate futures driving MC2 runs, and we tested our assumptions about fire suppression.

We evaluate the ability of our model to: 1) interpret vegetation modeling results and express risk over time in a useable way for managers and decision makers; 2) provide upper and lower bounds of that risk; 3) quantify uncertainty in a straightforward manner; 4) attribute uncertainty to its source; 5) attribute risk to its underlying drivers.

## Methods

### Study area

The study area (Fig 1) consists of the region of Oregon and Washington west of the Cascade Mountain Range crest that includes Coast Range, Klamath Mountains/California High North Coast Range, Willamette Valley, Puget Lowlands, Cascades, and North Cascades Level III Ecoregions [35]. This region is subject to strong coastal influence with mild, wet winters and warm dry summers.

### MC2 results used in this study

The protocol used to generate the MC2 results presented here was designed for an earlier project [14]. In this case, MC2 did not account for historical or future land use, nor past disturbances (pest outbreaks, diseases, or windthrow). Historical results (1895–2010) were obtained using PRISM [36] data and observed atmospheric CO_2_ concentrations as drivers. Our baseline period was 1971–2000. The vegetation model was run twice, once with fire suppression (FS) and once without (NFS—no fire suppression).

**Fig 2:**
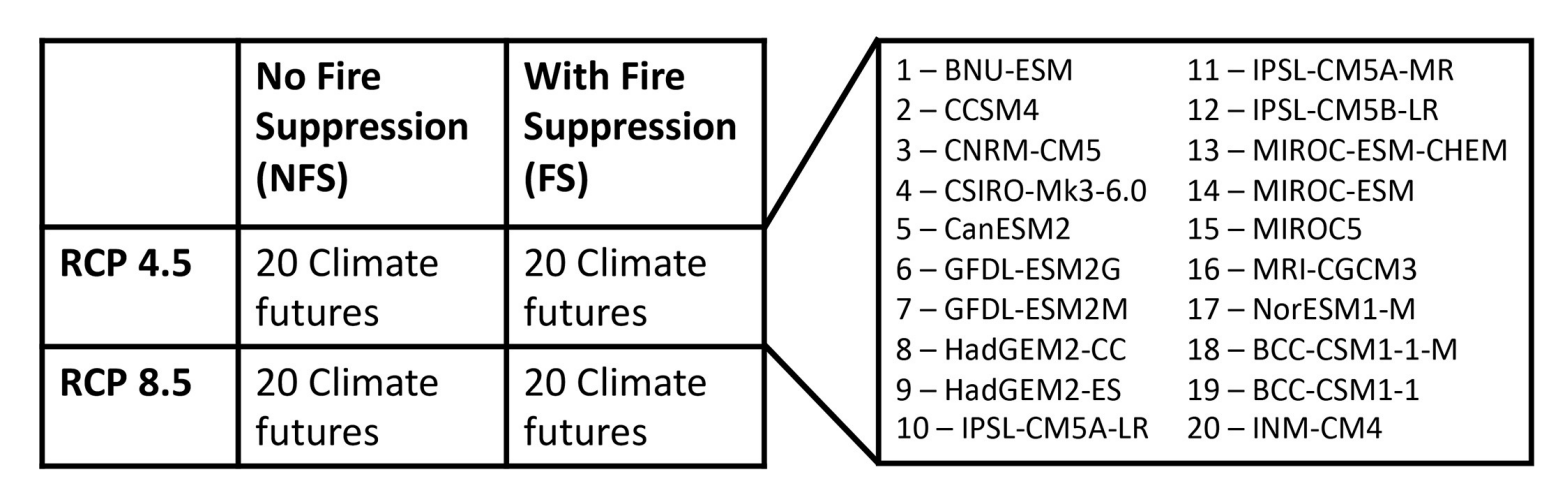
Model scenarios. Schematic of scenario and GCM/ESM climate driver combinations used to produce MC2 results used in this study.

Our future scenarios included either FS or NFS, with either Representative Concentration Pathway (RCP) 4.5 or 8.5 CO_2_ concentrations. For each of those scenarios, MC2 was run with 20 different climate futures from different Climate Model Intercomparison Project Phase 5 (CMIP5) [37] General Circulation Models (GCMs) or Earth System Models (ESMs; Fig 2). MC2 results were summarized over three time periods: early 21^st^ c. (2011–2030), mid 21^st^ c. (2036–2065), and late 21^st^ c. (2071–2099). We refer to one set of 20 MC2 results for one scenario and one future time period as an *ensemble* of results.

### EEMS fuzzy logic modeling

**Fig 3:**
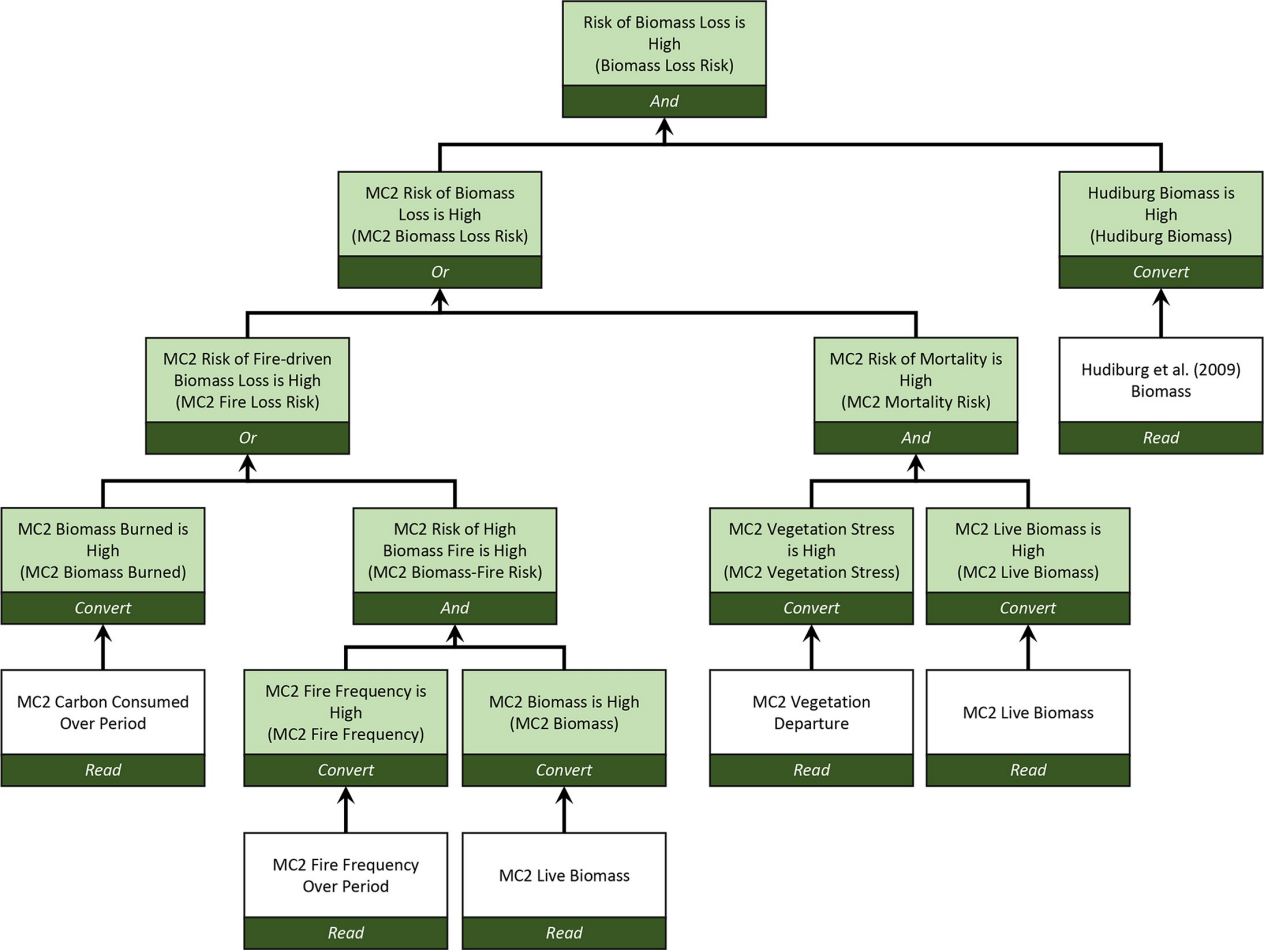
Logic tree for *Biomass Loss Risk* (formally *Risk of Biomass Loss is High*) EEMS model. Each model node (box) represents a spatial data layer (map). Unshaded nodes represent input data layers. Shaded nodes represent data layers with normalized variable values. Labels are formal fuzzy logic statements with informal index labels in parentheses.

The Environmental Evaluation Modeling System (EEMS) [38] is a fuzzy logic [39–40] modeling platform designed to inform answers to management questions. A model is represented by a logic tree, with each node corresponding to a displayable spatial layer or map (e.g. Fig 3). The bottom-most nodes in the tree represent input data layers. Each input layer is first normalized (0 to 1 for this study) to produce a node representing its level of agreement with a user-defined statement. For example, a fuel load metric might be mapped to the statement *Simulated Live Biomass is High* using user-defined thresholds to characterize *High*. Normalized values are combined into higher level nodes using fuzzy logic operators that evaluate the relationship between two or more datasets to another statement. For example, data for *Simulated Live Biomass is High* might be combined with data for *Vegetation Stress is High* to create a resulting node for *Mortality Risk is High*. In a complete model, nodes are repeatedly combined to produce a final, top-level node that informs the original management question.

Formally, each node in a fuzzy logic model corresponds to a factual statement, and the values for the node (the normalized values described above) are the values for the statement’s *fuzzy truth*. Fuzzy truths range from 0 for *fully false* to 1 for *fully true*. Values between 0.0 and 0.5 are considered *partially false*, 0.5 is *neither true nor false*, and values between 0.5 and 1.0 are *partially true*. Informally, values in the nodes are considered as indices for the attribute associated with the factual statement. For example, a fuzzy value for *Vegetation Stress is High* might be referred to simply as the level of *Vegetation Stress* from low (0) to high (1). We use the informal node labels hereafter.

**Fig 4:**
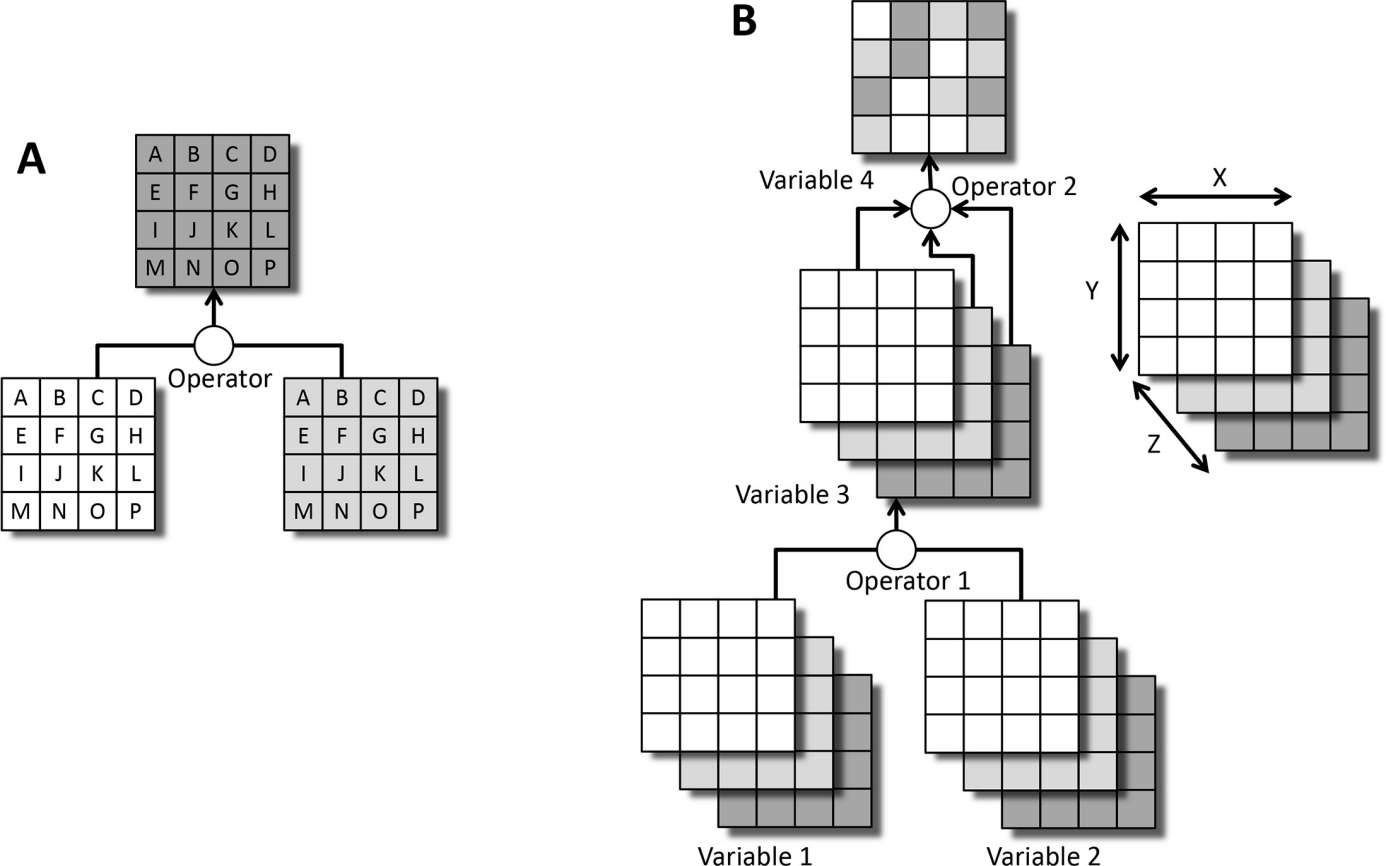
Operations in EEMS. A) Reporting units (grid cells in this example) in all data layers must correspond to one another. Fuzzy logic operators use the content of matching reporting units in different layers, but not between reporting units within the same layer. B) Two methods of applying fuzzy logic operations in the extended version of EEMS. Three-dimensional variables 1 and 2 are combined by operator 1 to produce the three-dimensional variable 3. Operator 2 uses values across the Z dimension of variable 3 to produce two-dimensional variable 4. In this study, the Z dimension corresponds to the 20 members of an ensemble.

The spatial datasets used in an EEMS model must share the same extent, projection, and reporting units (normally either polygons, or grid cells as in this study). Operations are performed using corresponding reporting units from different data layers (Fig 4A). Reporting units within layers are treated independently of one another and do not influence each other’s values.

The EEMS fuzzy logic operators used in this project are *And* (minimum value of the inputs), *Or* (maximum value of the inputs), and *Union* (mean value of the inputs). With the *And* and *Or* operators a reporting unit’s result value comes from only of the input values (unless multiple input values yield the same minimum value (for *And*) or maximum value (for *Or*)). For example, if corresponding cells from nodes A and B have values of 0.3 and 0.5, and these nodes are inputs to the *And* operator to produce node C, C’s value for the corresponding cell would be 0.3 and would come from only the cell in node A. A result of this is that the values in cells of a node produced by *And* or *Or* can be attributed to their source node.

### Decision support modeling

We created an EEMS decision support model (Fig 3) to evaluate the combined *Biomass Loss Risk* (formally, *Risk of Biomass Loss is High*) from fire and climate maladaptation using MC2 ensemble results and aboveground biomass simulated by [34] (hereafter, Hudiburg). For modeled risk to be considered high, the threat to a cell’s biomass–either from fire or modeled vegetation type departure from baseline vegetation type–must be high, and the biomass in the cell must also be high. High modeled biomass is insured by input variables in the *MC2 Biomass Loss Risk* branch of the model. Hudiburg’s biomass values are based on observed biomass measurements and their inclusion in the EEMS model serves to adjust *Biomass Loss Risk* down due to the legacy effects of disturbance and harvest.

Normalization of the datasets to obtain fuzzy values was done by establishing minimum (*fully false*) and maximum (*fully true*) thresholds and applying linear interpolation between thresholds, such that

fuzzyval=0 (1)

where *inputval* < *minthresh*

$$fuzzyval=\frac{\left(inputval-minthresh\right)}{\left(maxthresh-minthresh\right)}$$ (2)

where *minthresh* < = *inputval* < = *maxthresh*

fuzzyval=1 (3)

where *inputval* > *maxthresh* where *fuzzyval* is the normalized fuzzy value, *inputval* is the input (raw) data value, *minthresh* is the minimum threshold corresponding to the formal node statement, and *maxthresh* is the maximum threshold.

To normalize MC2 biomass and fire frequency values, we used the distribution of each variable over the study area during the baseline period. The 10^th^ percentile value for each variable was used as the minimum threshold and the 90^th^ percentile was used for the maximum threshold (Table 1). Similarly, we normalized Hudiburg’s biomass values and used the 10^th^ and 90^th^ percentile values from that data set. We calculated MC2 vegetation departure (a shift from the original modeled vegetation type to a new type) by comparing the cell’s modal vegetation type for a future period to its vegetation type for the baseline period. A departure value quantifying the level of disparity between past and future vegetation types was obtained from a lookup table based on expert opinion (S1 Table). To normalize the departure values and produce a data layer representing the overall vegetation stress level (*MC2 Vegetation Stress*) we used departure values of 0 and 3 for minimum and maximum thresholds respectively.

**Table 1:** EEMS conversion thresholds. Conversion thresholds used in the EEMS model to evaluate Biomass Loss Risk. Threshold values are based on the distribution of each variable except for vegetation type departures.

Variable	*Fully false* or minimum threshold	*Fully true* or maximum threshold	Comments
MC2 Biomass Burned	0 (g C m^-2^)	110 (g C m^-2^)	Threshold values from historical period distribution. False threshold 10^th^ percentile, True threshold 90^th^ percentile.
MC2 Fire Frequency	0.0 (decimal fraction)	1.0 (decimal fraction)	Fraction of years with fire. False threshold 10^th^ percentile, True threshold 90^th^ percentile.
MC2 Biomass	31572 (g C m^-2^)	73148 (g C m^-2^)	Threshold values from historical period distribution. False threshold 10^th^ percentile, True threshold 90^th^ percentile.
MC2 Live Biomass	5839 (g C m^-2^)	29387 (g C m^-2^)	Threshold values from historical period distribution. False threshold 10^th^ percentile, True threshold 90^th^ percentile.
Hudiburg Biomass	4053 (g C m^-2^)	21844 (g C m^-2^)	False threshold 10^th^ percentile, True threshold 90^th^ percentile.
MC2 Vegetation Stress	0 (departure value)	3 (departure value)	Level of vegetation departure from historical based on expert opinion (S1 Table).

(g: gram; C: carbon; m: meter)

### Uncertainty Analysis

We characterized uncertainty by first calculating the variability of *Biomass Loss Risk* values spatially across each ensemble of results. Results from each ensemble of 20 MC2 runs were combined into 3-dimensional datasets with ensemble members comprising the third dimension (Fig 4B). *Biomass Loss Risk* was calculated independently for each ensemble member. An extended version of EEMS was used to produce a data layer for each of the minimum, maximum, and mean fuzzy values (Fig 4B), bracketing the variability. The fuzzy value *High Variability* (formally, *Variability is High*) was calculated for each cell in each ensemble by converting standard deviations into fuzzy space using the minimum possible standard deviation (0) as the false threshold and the maximum possible standard deviation (0.5) as the true threshold.

We characterized the non-spatial uncertainty between members of each ensemble using box and whisker plots of their area-weighted means for *Biomass Loss Risk*. Plots for all scenarios within a time period are displayed together for inter-scenario comparison.

To determine whether climate futures’ annual temperature and/or precipitation are tightly coupled with *MC2 biomass loss risk*, we evaluated the relationships between those 3 variables. First, we compared each ensemble member’s area-weighted mean change in temperature from the baseline period against its change in precipitation. Secondly, we compared each ensemble member’s contribution to an ensemble’s fraction of area matching the maximum *MC2 Biomass Loss Risk* vs its fraction of area matching the minimum. A visual comparison of an ensemble member’s position in the first graph to its position in the second graph illustrates the strength of relationship between these two measures.

### Drivers of biomass loss risk

The *Or* operator in the model node *MC2 Biomass Loss Risk* (Fig 3) takes the maximum values of the two inputs, one corresponding to the simulated biomass lost by fire from the MC2 model, the other corresponding to the risk of mortality due to vegetation shift (not due to fire) as simulated by MC2. For each ensemble, we took the ensemble mean for each of *MC2 Fire Loss Risk*, *MC2 Mortality Risk*, and *MC2 Fire Loss Risk* minus *MC2 Mortality Risk* to show which factor most strongly drives *MC2 Biomass Loss Risk*. Absolute difference values are greatest where one factor produces a high risk and the other produces a low risk. These results reflect the contribution to *Biomass Loss Risk* from MC2 results without the contribution from *Hudiburg Biomass*.

We characterized the influence of fire versus that of vegetation shift over the study area. For each ensemble member, we compared the fraction of the area for which mortality due to vegetation shift was the dominant driver of the risk to lose biomass vs the fraction of area where fire was the main driver of risk. Grid cells with a zero risk value were not considered.

## Results

In this manuscript, we present detailed results for the RCP 8.5 / NFS / 2071–2099 time period ensemble and summary results from other ensembles. Detailed results from other ensembles are in supplemental materials.

### Decision support modeling

**Fig 5:**
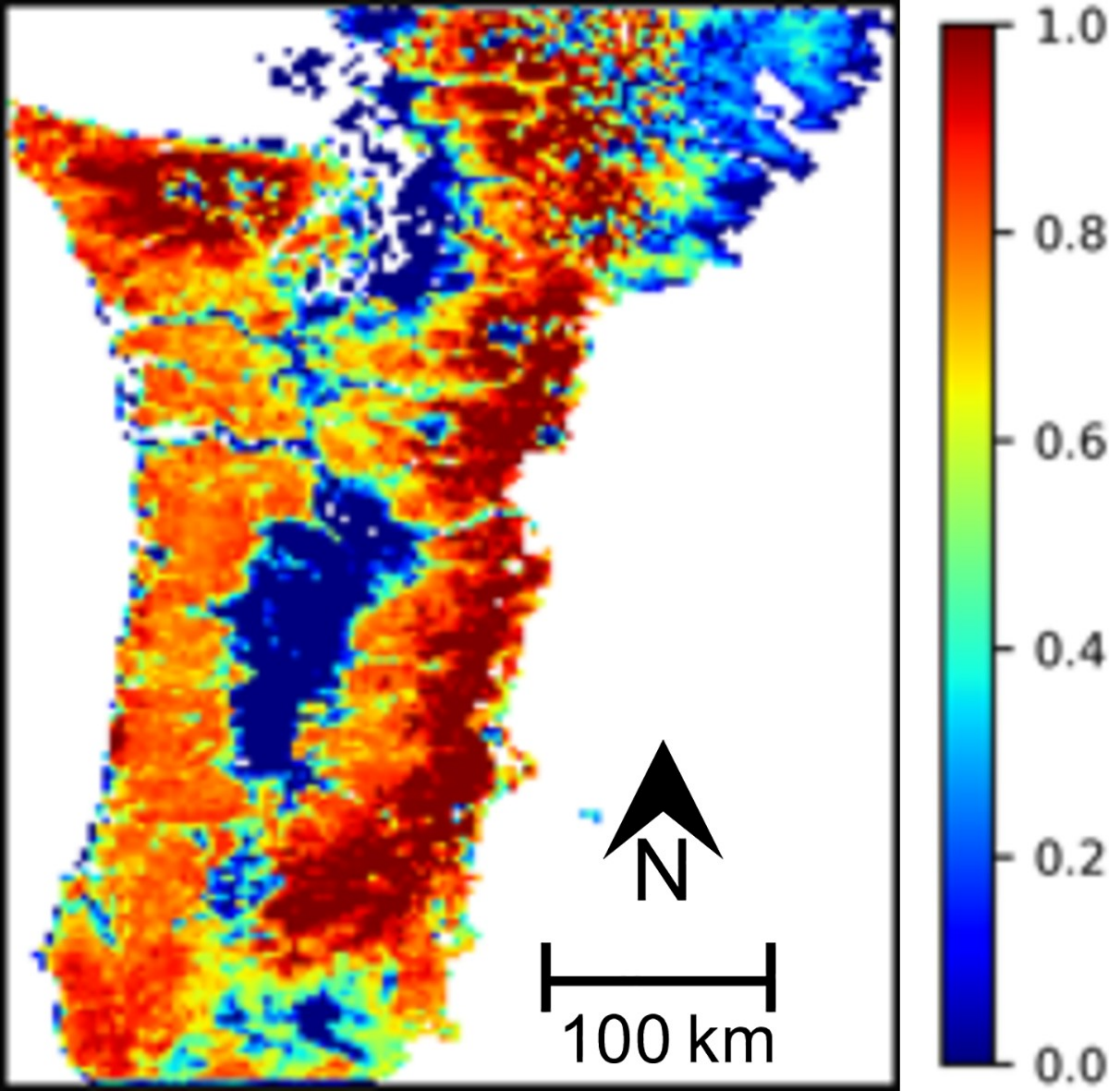
Hudiburg Biomass. 11% of the area has a value of 0.

We used normalized biomass values from Hudiburg (Fig 5) for all our EEMS model runs. Biomass is highest in the Cascade Mountains and in the Olympic Peninsula, and lowest around Puget Sound, on the east side of the Northern Cascades, throughout the Willamette Valley, and in southern Oregon around the cities of Roseburg, Medford, and Ashland. Eleven percent of the study area is assumed to have zero biomass.

**Fig 6:**
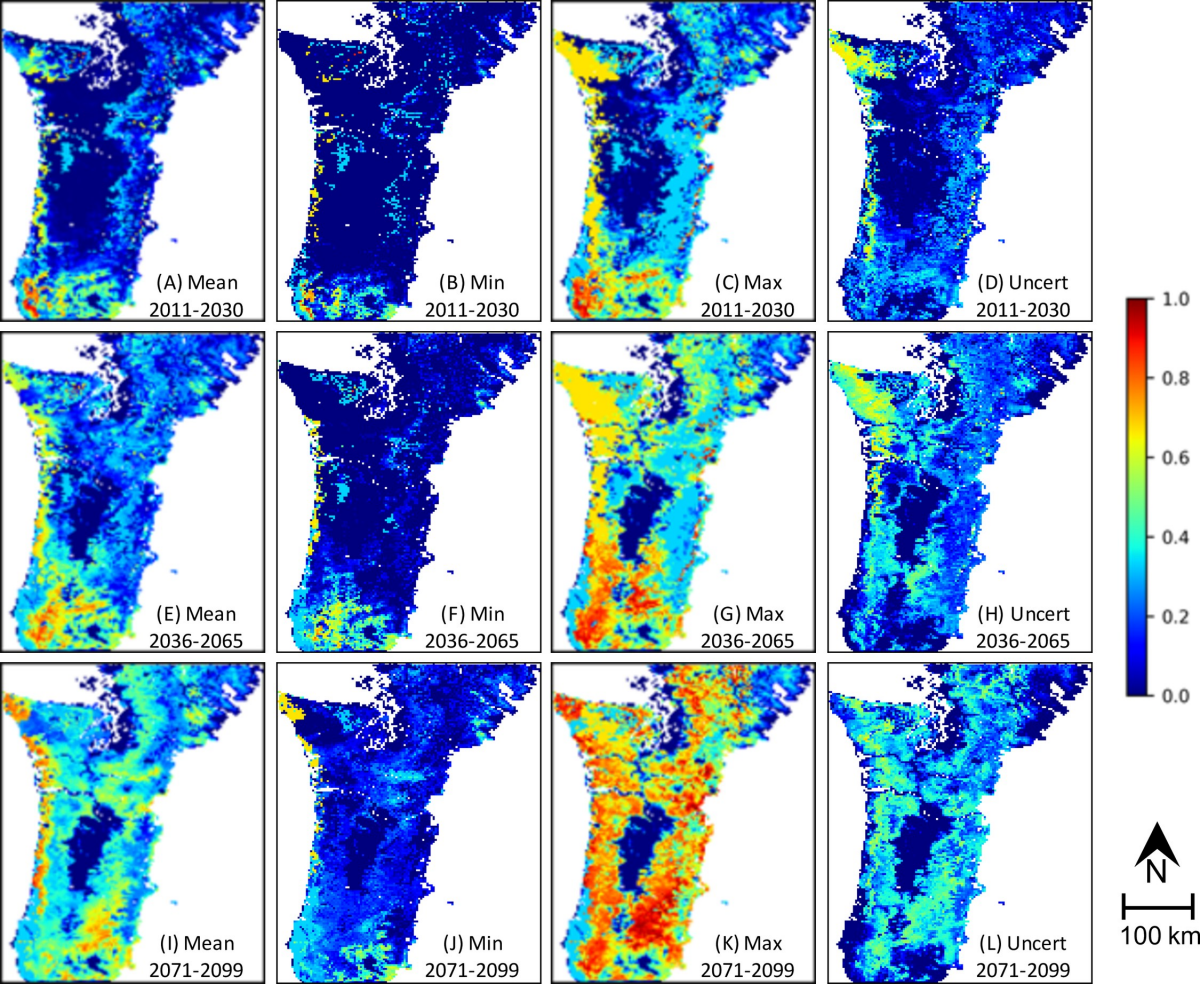
Maps of *Biomass Loss Risk* from EEMS model for the RCP 8.5 NFS scenario. Figure rows include the mean, minimum, maximum, and uncertainty representation for one time period. (min: minimum; max: maximum; uncert: uncertainty).

*Biomass Loss Risk* is low in areas where *Hudiburg Biomass* is low (Figs 5–6 and S1–S3 Figs). For RCP 8.5 with or without fire suppression, mean and minimum values of *Biomass Loss Risk* are highest during the mid 21^st^ c. in the southern portion of the study area (Fig 6 and S3A, S3B, S3E, S3F, S3I, S3J Fig). For RCP 4.5, the trend is similar, but less pronounced (S1–S2 Figs A, B, E, F, I, J), and less apparent or absent for maximum values across all scenarios (Fig 6 and S1–S3 Figs C, E, K). Overall, the risk of biomass loss is higher in the southern portion of the study area and the Coast Range than in the Cascade Range (Fig 6 and S1–S3 Figs A, B, C, E, F, G, I, J, K).

**Fig 7:**
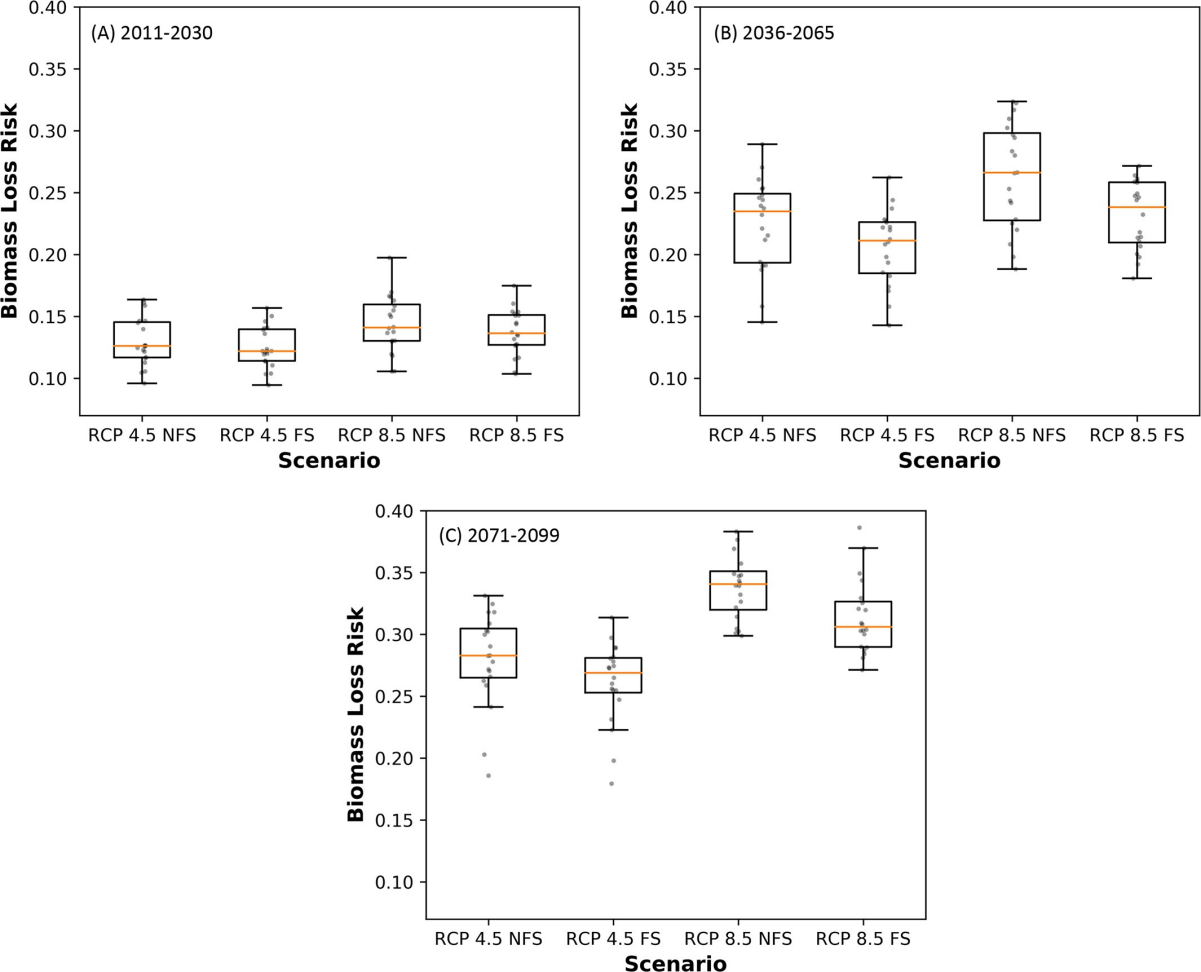
Distribution of area weighted mean values *Biomass Loss Risk* from EEMS model. Each point represents the area weighted mean of one ensemble member.

The area weighted mean of *Biomass Loss Risk* increases with time, is lower for RCP 4.5 than for RCP 8.5, and is slightly higher for NFS scenarios than for FS (Fig 7, Table 2). The range of values increases for all scenarios through time (Fig 7, Table 2).

**Table 2:** Regional values for *Biomass Loss Risk*. Mean, minimum, maximum, and uncertainty for area weighted mean of *Biomass Loss Risk* EEMS model.

	2011–2030			2036–2065			2071–2099		
	Mean	Range (min-max)	Uncertainty	Mean	Range (min-max)	Uncertainty	Mean	Range (min-max)	Uncertainty
**RCP 4.5, FS**	0.13	0.05–0.22	0.11	0.21	0.07–0.36	0.19	0.26	0.09–0.43	0.21
**RCP 4.5, NFS**	0.13	0.05–0.23	0.11	0.23	0.07–0.38	0.20	0.28	0.09–0.45	0.22
**RCP 8.5, FS**	0.14	0.05–0.24	0.12	0.23	0.08–0.39	0.19	0.32	0.12–0.51	0.22
**RCP 8.5 NFS**	0.15	0.06–0.27	0.14	0.27	0.09–0.42	0.20	0.34	0.14–0.53	0.23

(min: minimum; max: maximum)

### Uncertainty

Uncertainty is 0 where *Hudiburg Biomass* is 0, and is generally lower or higher corresponding to lower and higher values for *Biomass Loss Risk* (Fig 6 and S1–S3 Figs D, H, L). In the Olympic Peninsula, uncertainty is generally higher overall except near the end of the century for the RCP 8.5 scenarios. In the southeastern portion of the study area, uncertainty is low across all scenarios. Area weighted mean uncertainty is similar overall and increases with time (Table 2).

Between RCP 4.5 and RCP 8.5, uncertainty ranges from 0.01 to 0.09, increasing through time (Table 3). Between FS and NFS, uncertainty ranges from 0.00 to 0.03, with the lowest values for the early 21^st^ c. (Table 3).

**Table 3:** Area weighted mean of uncertainty for RCP 4.5 vs RCP 8.5 and FS vs NFS.

	2011–2030	2036–2065	2071–2099
**RCP 4.5 vs RCP 8.5, FS**	0.02	0.04	0.09
**RCP 4.5 vs RCP 8.5, NFS**	0.02	0.05	0.10
**FS vs NFS, RCP 4.5**	0.00	0.02	0.02
**FS vs NFS, RCP 8.5**	0.01	0.03	0.03

### Drivers of Results

**Fig 8:**
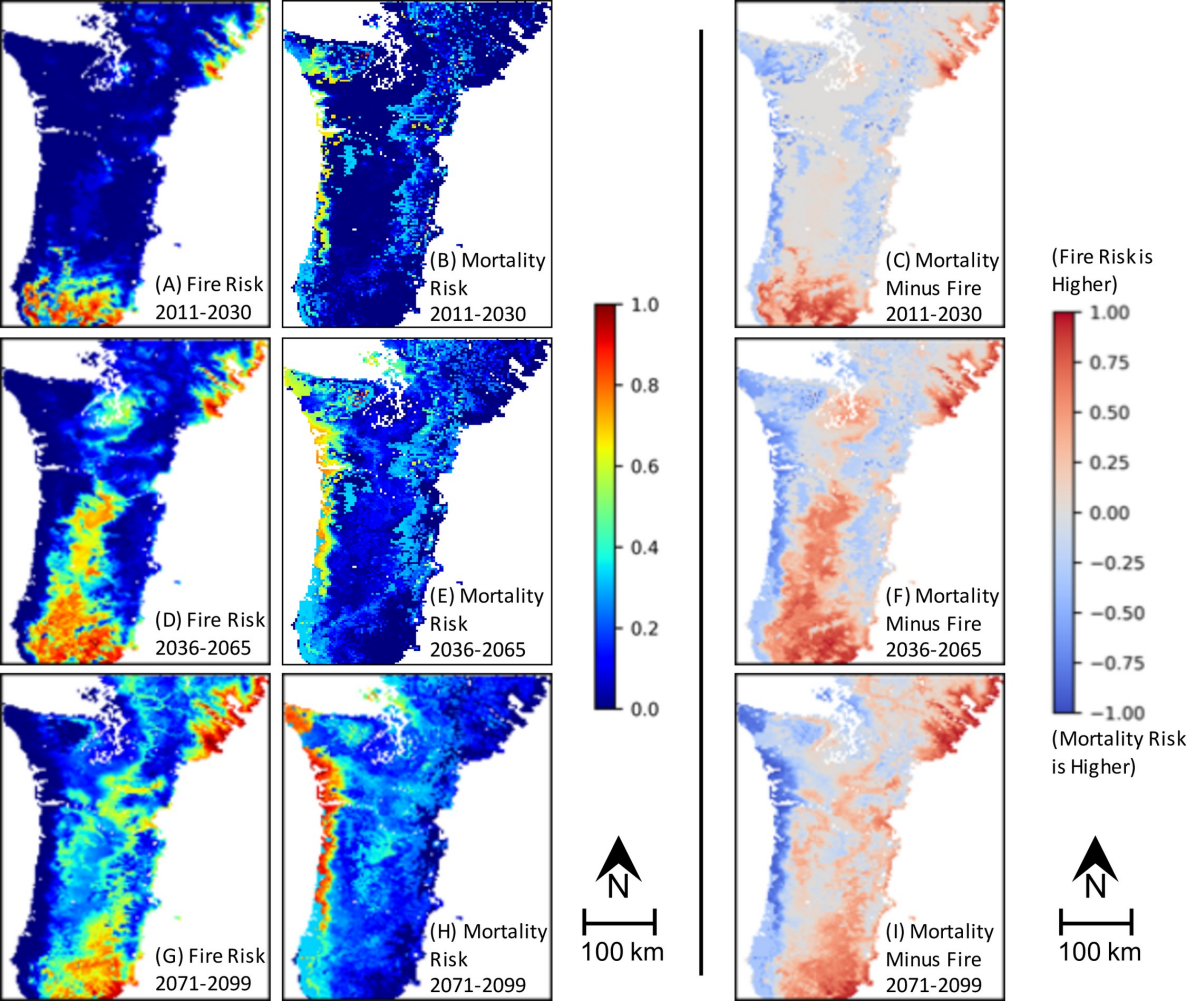
Drivers of *MC2 Mortality Risk*. Maps of *MC2 Fire Loss Risk* (A, D, G), *MC2 Mortality Risk* (B, E, H), and *MC2 Fire Loss Risk* minus *MC2 Mortality Risk* (C, F, I) from the EEMS model for the RCP 8.5 NFS scenario. Figure rows represent time periods.

*MC2 Fire Loss Risk* (Fig 8 and S4–S6 Figs A, D, F) is greatest in the southern portion of the study region and generally expands through time north through the Willamette Valley and Puget Trough in the center of the region, east and west from the center into the foothills of the Coast and Cascade mountain ranges, and also on the northeast edge of the study region. The expansion into the Coast and Cascade ranges is greater under NFS than FS and markedly greater under RCP 8.5 than under RCP 4.5, with expansion towards the Cascade crest in the late 21^st^ c. (Fig 8 and S6 Fig. G). *MC2 Fire Loss Risk* falls in the southern and eastern portions of the study area under RCP 8.5 in the late 21^st^ c. (Fig 8 and S6 Fig. G).

*MC2 Mortality Risk* (Fig 8 and S4–S6 Figs B, E, H) is greatest along the coast, somewhat high in the Olympic Peninsula of northwestern Washington, and expands into the foothills of the Cascades and the central portion of the study region through time. It is greater under RCP 8.5 than RCP 4.5, and is virtually unaffected by +/- fire suppression.

The general spatial separation of high *MC2 Fire Loss Risk* from high *MC2 Mortality Risk* (Fig 8 and S4–S6 Figs A, B, D, E, G, H) is reflected in the driver difference maps (Fig 8 and S4–S6 Figs C, F, I). The southern portion and northeastern corner of the study area are driven by fire whereas the coast and Olympic Peninsula are driven by mortality. Under RCP 4.5, mortality drives *MC2 Biomass Loss Risk* more strongly in the Cascades (S4–S5 Figs C, F, I). However, under RCP 8.5, the stronger driver shifts from mortality to fire in the Cascades in the late 21^st^ c (Fig 8 and S6 Fig. C, F, I).

**Fig 9:**
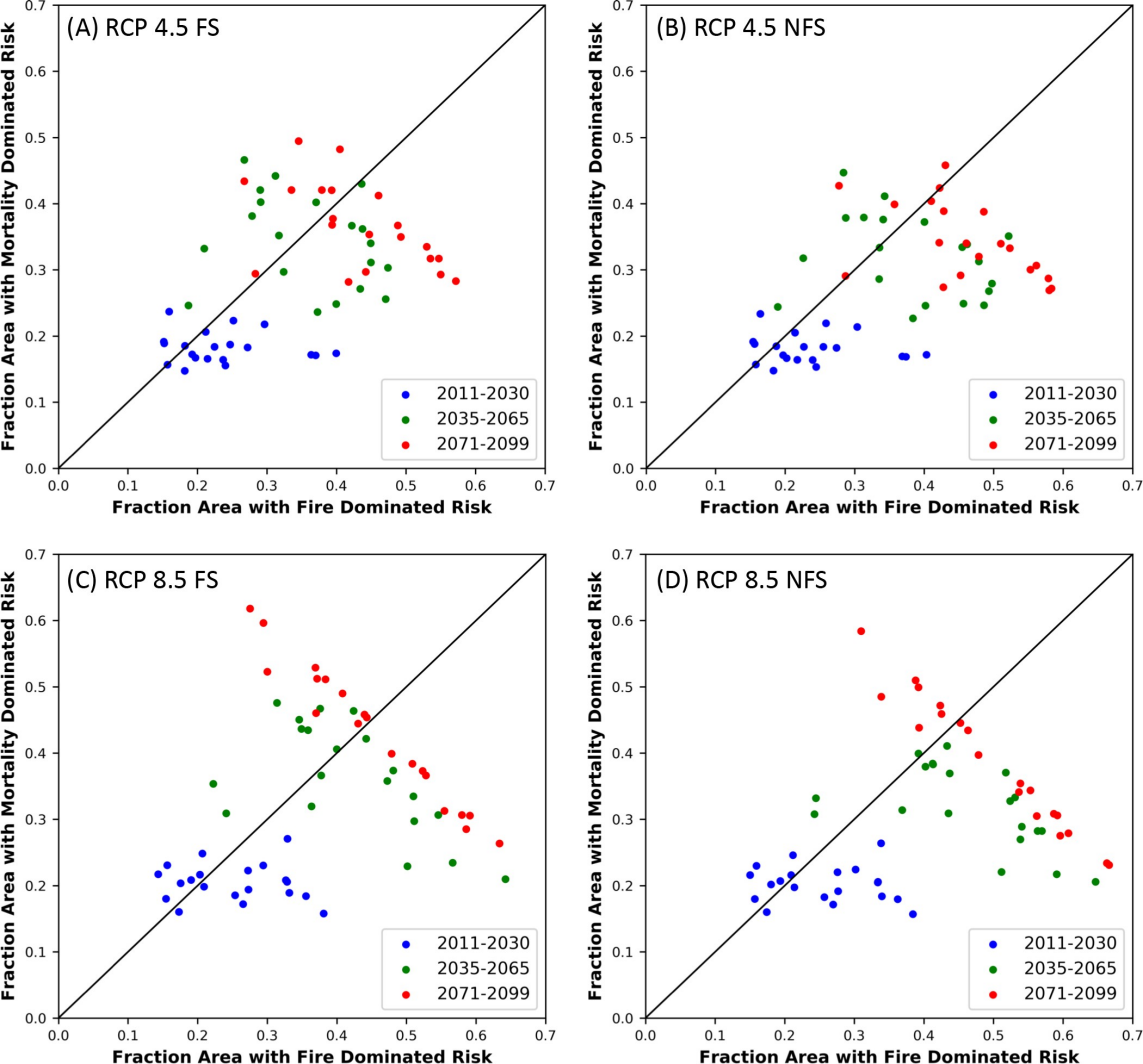
Drivers of *MC2 Biomass Loss Risk*. Fraction of the study area at risk driven by vegetation shifts (*MC2 Mortality Risk*) versus driven by fire (*MC2 Fire Loss Risk*) in the EEMS node *MC2 Biomass Loss Risk*.

The fraction of area with 0 *MC2 Biomass Loss Risk* is somewhat greater for RCP 4.5 scenarios than for RCP 8.5, and declines through time, with a minimum of 11% for any ensemble member (Table 4). Fire (*MC2 Fire Loss Risk*) contributes more to the risk of losing biomass (*Biomass Loss Risk*) than does vegetation shift (*Mortality Risk*) for all ensembles with the exception of RCP 8.5, FS, 2071–2099 (Table 5, Fig 9). The difference is generally smaller for FS scenarios than for NFS scenarios.

**Table 4:** Area-weighted summary of *MC2 Biomass Loss Risk* drivers. Fraction of area with no risk of losing biomass, risk driven by either fire (*MC2 Fire Loss Risk*) or vegetation shift (*MC2 Mortality Risk*) in the EEMS *MC2 Biomass Loss Risk* model node.

	2011–2030			2036–2065			2071–2099		
	Zero risk (min-max (mean))	Fire-dominated (min-max (mean))	Mortality-dominated (min-max (mean))	Zero risk (min-max (mean))	Fire-dominated (min-max (mean))	Mortality-dominated (min-max (mean))	Zero risk (min-max (mean))	Fire-dominated (min-max (mean))	Mortality-dominated (min-max (mean))
**RCP 4.5 FS**	0.43–0.69 (0.58)	0.15–0.40 (0.24)	0.15–0.24 (0.18)	0.13–0.57 (0.30)	0.19–0.47 (0.36)	0.24–0.47 (0.34)	0.11–0.42 (0.20)	0.27–0.57 (0.43)	0.28–0.49 (0.37)
**RCP 4.5 NFS**	0.42–0.69 (0.58)	0.15–0.40 (0.24)	0.15–0.23 (0.18)	0.13–0.57 (0.29)	0.19–0.52 (0.38)	0.23–0.45 (0.32)	0.11–0.42 (0.20)	0.28–0.58 (0.46)	0.27–0.46 (0.34)
**RCP 8.5 FS**	0.40–0.67 (0.54)	0.14–0.38 (0.25)	0.16–0.27 (0.20)	0.11–0.45 (0.21)	0.22–0.64 (0.42)	0.21–0.48 (0.36)	0.10–0.18 (0.12)	0.28–0.63 (0.45)	0.26–0.62 (0.43)
**RCP 8.5 NFS**	0.40–0.67 (0.54)	0.15–0.38 (0.26)	0.16–0.26 (0.20)	0.11–0.45 (0.21)	0.24–0.65 (0.47)	0.21–0.41 (0.32)	0.10–0.18 (0.12)	0.31–0.67 (0.50)	0.23–0.58 (0.39)

(min: minimum; max: maximum)

**Table 5:** Per ensemble drivers of *MC2 Biomass Loss Risk*. Driving factor of the risk of losing biomass illustrated by the number of ensemble members for which either fire (*MC2 Fire Loss Risk*) or simulated vegetation shifts (*MC2 Mortality Risk*) drive the risk of biomass loss for *MC2 Biomass Loss Risk*.

	2011–2030		2036–2065		2071–2099	
	Fire dominated (count)	Mortality dominated (count)	Fire dominated (count)	Mortality dominated (count)	Fire dominated (count)	Mortality dominated (count)
**RCP 4.5 FS**	16	4	11	9	13	7
**RCP 4.5 NFS**	17	3	13	7	15	5
**RCP 8.5 FS**	13	7	11	9	9	11
**RCP 8.5 NFS**	13	7	17	3	13	7

**Fig 10:**
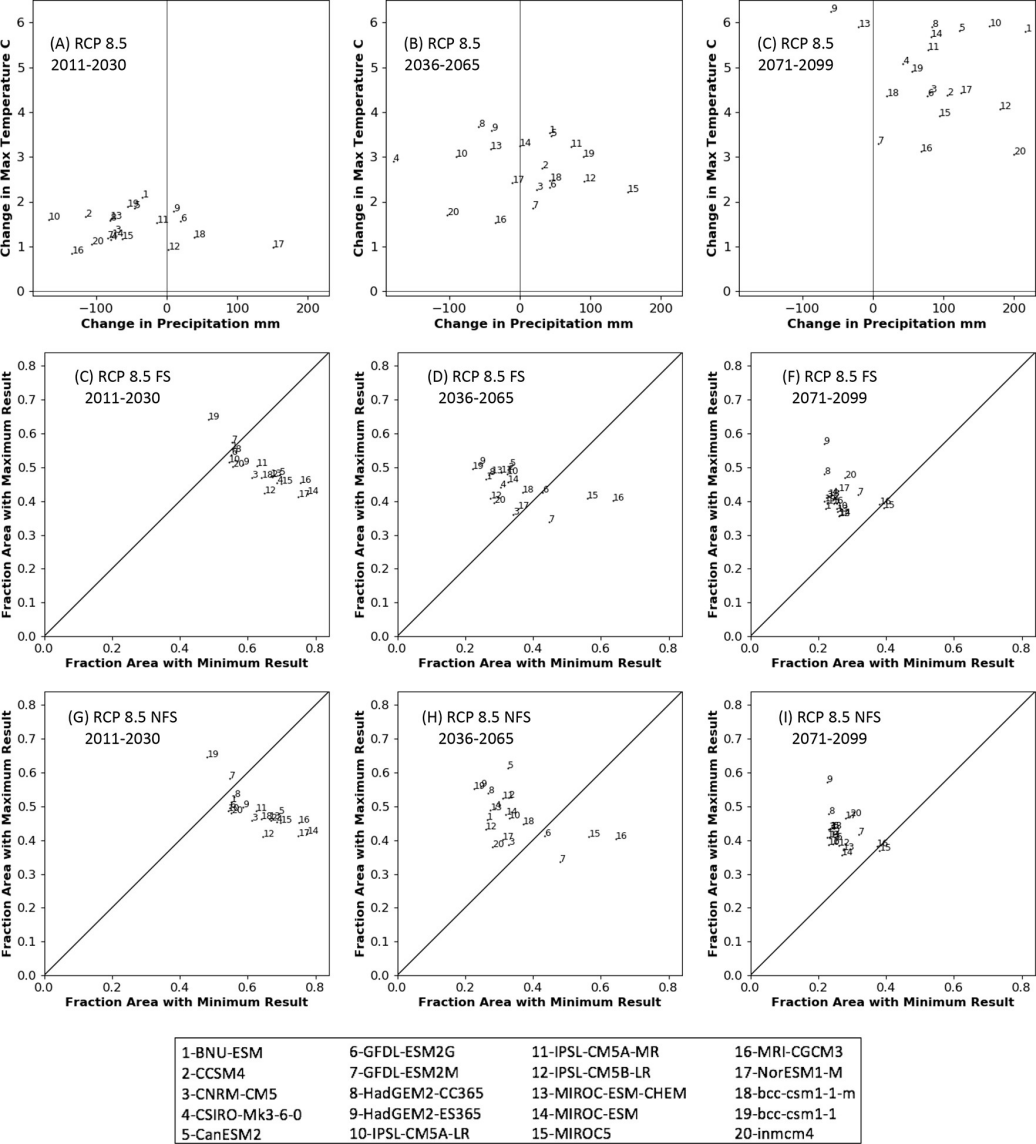
Relationship between climate change summary and MC2 Biomass Loss Risk. Change (1971–2000 vs future time period) in average maximum temperature vs change in average annual precipitation for each of the 20 RCP 8.5 climate futures (A-C), and fraction of the simulated area with maximum and minimum values *MC2 Biomass Loss Risk* for the RCP 8.5 FS scenario (D-F) and the NFS scenario (G-I). In graphs D-I, a point above the 45° line indicates that the results of the MC2 run driven by that climate future showed a greater number of high vs low values of *MC2 Biomass Loss Risk* over more of the study area. Points below the 45° line indicate that MC2 results showed a greater number of low vs high values over more of the area. (mm: millimeters).

Ensemble members with the greatest (or least) change in annual temperature generally do not correspond to climate futures responsible for the largest area of maximum (minimum) risk of biomass loss (Fig 10 and S7 Fig.). One exception is under HadGEM2-ES365 (model 9) which drives the greatest change in temperature for 2071–2099 under both RCP 4.5 and RCP 8.5 and causes the greatest simulated area at risk in the node *Maximum Biomass Loss Risk*. Over time, the ratio of the number of climate models causing the largest vs the smallest areas at risk of losing biomass (*Biomass Loss Risk)* increases substantially (2-5/x models for 2011–2030 to 17-19/x for 2071–2099).

## Discussion

### Context

We compared our method of presenting uncertainty with those from seven studies covering our study area using inputs from climate models. Climate-based uncertainty was handled in a variety of ways. [15] used the average results from two GCMs, predicating their results on the correctness of those GCMs and the CO_2_ projections driving them. [16] used an ensemble mean of results from 17 GCMs as a means of defining a consensus future climate before using that climate in their model. Many studies presented graphs and/or tables of region-summarized values of selected drivers and results [14, 19–20, 22, 25, 33]. Several presented sets of maps allowing visual comparisons of result variation [14, 19–20, 25, 33], but only two presented spatial uncertainty. [19] classified risk to Douglas fir in terms of the percentage of models agreeing or disagreeing on its occurrence, and [20] mapped the number of models agreeing on the direction of change in ecosystem carbon, burn area, and vegetation type. To our knowledge, ours is the first study in this region to provide a detailed, quantified, spatial measure of climate-based uncertainty for modeled future vegetation.

### Limitations

An ensemble mean provides a single measure of risk for the ensemble, however climate models driving the results may not be completely independent of one another [41]. Weighting results based on the similarities of the underlying climate models could adjust for this but understanding the provenance of many climate models can be onerous.

We did not account for the uncertainty resulting from assumed ignitions or the built-in CO_2_ fertilization effect in MC2. The consequences of these assumptions on MC2 fire and carbon dynamics results were found to be substantial [25], but including those uncertainties was beyond the goal of this study. Likewise, we did not incorporate the uncertainty in Hudiburg’s [34] data due to the study’s limited scope.

### General implications

Our results show the risk of biomass loss generally increasing with time in current high biomass areas within the study region. The pattern of increased fire-driven risk through time is generally south to north and upslope as fires become more frequent due to increasing temperatures. Mortality-driven risk increases along the coast where vegetation becomes maladapted to warming and where coastal climate influences reduce fire risk.

Changes in biomass are directly related to ecosystem services such as timber production, carbon sequestration, wildlife habitat provision, recreational opportunities, and fresh water quality [42]. Thinning, prescribed fire, and suppression can mitigate fire risk, however each of these actions has associated economic and other costs [43–44]. Thinning may increase forest resistance and resilience to drought, however, it may make forests less resistant and resilient as forests age [45]. When physiological processes cannot be buffered against environmental variability, maladaptation leads to mortality [18]. Maintaining biomass in forested areas under climate change-induced maladaptation may depend on management strategies such as sourcing seeds and species from better climatically suited sources (i.e. assisted migration) [46–47]. Tools to help mangers implement such strategies have been developed (e.g. Seedlot Selection Tool, https://seedlotselectiontool.org/sst/).

### Management and planning

Our results provide managers with spatial datasets representing three aspects of the *Biomass Loss Risk* metric. The mean value of biomass lost across all climate futures provides an overall idea of potential magnitude of loss while minimum and maximum values bracket the range, suggesting limits for management alternatives. Uncertainty quantifies the variability of the model results. Land management takes place at multiple scales, with planning and assessment at the national or regional level and implementation at more local levels [48]. Using terminology appropriate for managers such as *risk* and *uncertainty*, as well as using a spatial scale appropriate for local information makes our results appropriate for local planning.

Environmental models are often found to be insufficiently accurate to use as forecasts [31]. However, they provide insights and scenarios useful for scenario planning [31] and are useful for decreasing uncertainty rather than making predictions [28]. Our work is intended to be viewed within this context, providing one set of results with as much clarity as possible regarding uncertainty, sources of uncertainty, and drivers of risk.

Process-based models, such as MC2, are considered more limited than empirical models in quantifying uncertainty [28], thus limiting their usefulness in management planning. Our method alleviates this limitation, making it easier for managers to use process-based models in their decision making.

It has been suggested that when a range of future possibilities is needed for planning, selecting the most extreme climate projections (e.g. warmest, coolest, wettest, driest) as inputs to ecological models provides brackets for the needed answers [28]. The lack of correspondence we found between the most extreme climate futures and their influence on minimum or maximum risk indicate that simple metrics for climate extremes are not sufficient for bracketing our model results. In a process-based model such as MC2, seasonal patterns and extreme events that are not reflected in annual values or averages over multi-year time periods have the potential to strongly affect fire and vegetation trajectories. Finding climate metrics that predict the most extreme results would be challenging, if not impossible, due to complex interactions within the model. While culling input datasets from GCMs and ESMs that perform poorly in the region may be required to reduce uncertainty [49], culling less extreme climate futures may inadvertently reduce the desired range of results.

## Conclusions

Fuzzy logic modeling has been used in a variety of ecological modeling applications [50], including species distribution (e.g. [51]), habitat mapping (e.g. [52]), water quality (e.g. [53]), wildfire risk (e.g. [54]), and the human valuation of natural elements (e.g. [55]). Managing forests in light of climate change requires understanding climate’s potential effects on not only forests, but also industries and communities [26].

Our model may prove useful to managers by itself, but it has the potential to provide greater utility when combined with other metrics reflecting landscape condition, status, and value. The modular nature of the EEMS framework would allow our model to be easily combined into new models. Ignition probabilities, fire spread probabilities, and fire refugia data [56–58] could be added to provide greater detail for fire risk. Submodels of climate refugia related to microclimate and enduring landscape features [59–60] could provide more realism for mortality-based risk. Combining our risk model with submodels for current habitat quality (e.g. [61]), connectivity corridors [62], and species presence or absence could help guide management conservation decisions. Similarly, incorporating risk with submodels for economic, social, and cultural values could help managers with biocultural approaches to conservation [63]. Stakeholder input and expert opinion can be used to parameterize these models so that they precisely reflect management concerns. It is our hope that this model and this methodology can contribute to sound decision making for a wide variety of purposes in our study region and beyond.

## Supporting information

S1 Table Lookup table for vegetation type differences. (DOCX)

S1 Fig Maps of *Biomass Loss Risk* from EEMS model for the RCP 4.5 FS scenario. Figure rows include the mean, minimum, maximum, and uncertainty representation for one time period. (min: minimum; max: maximum; uncert: uncertainty).
(TIF)

S2 Fig Maps of *Biomass Loss Risk* from EEMS model for the RCP 4.5 NFS scenario. Figure rows include the mean, minimum, maximum, and uncertainty representation for one time period. (min: minimum; max: maximum; uncert: uncertainty).
(TIF)

S3 Fig Maps of *Biomass Loss Risk* from EEMS model for the RCP 8.5 FS scenario. Figure rows include the mean, minimum, maximum, and uncertainty representation for one time period. (min: minimum; max: maximum; uncert: uncertainty).
(TIF)

S4 Fig Drivers of *MC2 Mortality Risk*. Maps of *MC2 Fire Loss Risk* (A, D, G), *MC2 Mortality Risk* (B, E, H), and *MC2 Fire Loss Risk* minus *MC2 Mortality Risk* (C, F, I) from EEMS model for the RCP 4.5 FS scenario. Figure rows represent time periods.
(TIF)

S5 Fig Drivers of *MC2 Mortality Risk*. Maps of *MC2 Fire Loss Risk* (A, D, G), *MC2 Mortality Risk* (B, E, H), and *MC2 Fire Loss Risk* minus *MC2 Mortality Risk* (C, F, I) from EEMS model for the RCP 4.5 NFS scenario. Figure rows represent time periods.
(TIF)

S6 Fig Drivers of *MC2 Mortality Risk*. Maps of *MC2 Fire Loss Risk* (A, D, G), *MC2 Mortality Risk* (B, E, H), and *MC2 Fire Loss Risk* minus *MC2 Mortality Risk* (C, F, I) from EEMS model for the RCP 8.5 FS scenario. Figure rows represent time periods.
(TIF)

S7 Fig Relationship between climate change summary and MC2 Biomass Loss Risk. Change (1971–2000 vs future period) in average maximum temperature vs change in average annual precipitation for each of the 20 RCP 4.5 climate futures (A-C), and fraction of the simulated area with maximum and minimum values MC2 *Biomass Loss Risk* for the RCP 4.5 FS scenario (D-F) and the NFS scenario (G-I). In graphs D-I, a point above the 45° line indicates that the results of the MC2 run driven by that climate future showed a greater number of high vs low values of biomass loss over more of the study area. Points below the 45° line indicate that MC2 results showed a greater number of low vs high values over more of the area. (mm: millimeters).
(TIFF)
