# Supplementary material for: A fuzzy logic decision support model for climate-driven biomass loss risk in western Oregon and Washington
Source: PLoS One. 2019 Oct 25;14(10):e0222051. doi: 10.1371/journal.pone.0222051 (PMC6814215; doi:10.1371/journal.pone.0222051)
Supplement: S1 Table — (DOCX) [file pone.0222051.s001.docx]

|  | **Undefined** | **Barren** | **Tundra** | **Taiga Tundra** | **Boreal Evergreen Needleleaf Forest** | **Boreal Mixed Woodland** | **Subalpine** | **Maritime Evergreen Needleleaf Forest** | **Temperate Evergreen Needleleaf Forest** | **Temperate Deciduous Broadleaf Forest** | **Temperate Cool Mixed Forest** | **Temperate Warm Mixed Forest** | **Temperate Evergreen Needleleaf Woodland** | **Temperate Deciduous Broadleaf Woodland** | **Temperate Cool Mixed Woodland** | **Temperate Warm Mixed Woodland** | **Temperate Shrubland** | **Temperate Grassland** | **Temperate Desert** |
| --- | --- | --- | --- | --- | --- | --- | --- | --- | --- | --- | --- | --- | --- | --- | --- | --- | --- | --- | --- |
| **Undefined** | 0 | 5 | 5 | 5 | 5 | 5 | 5 | 5 | 5 | 5 | 5 | 5 | 5 | 5 | 5 | 5 | 5 | 5 | 5 |
| **Barren** | 5 | 0 | 1 | 2 | 5 | 4 | 5 | 5 | 5 | 5 | 5 | 5 | 4 | 4 | 4 | 4 | 3 | 2 | 1 |
| **Tundra** | 5 | 1 | 0 | 1 | 3 | 2 | 4 | 5 | 5 | 5 | 5 | 5 | 5 | 5 | 5 | 5 | 5 | 5 | 5 |
| **Taiga Tundra** | 5 | 2 | 1 | 0 | 2 | 1 | 3 | 5 | 5 | 5 | 5 | 5 | 5 | 5 | 5 | 5 | 5 | 5 | 5 |
| **Boreal Evergreen Needleleaf Forest** | 5 | 5 | 3 | 2 | 0 | 2 | 1 | 2 | 2 | 4 | 3 | 4 | 2 | 4 | 3 | 5 | 5 | 5 | 5 |
| **Boreal Mixed Woodland** | 5 | 4 | 2 | 1 | 2 | 0 | 2 | 4 | 3 | 3 | 2 | 3 | 2 | 2 | 1 | 2 | 3 | 4 | 5 |
| **Subalpine** | 5 | 5 | 4 | 3 | 1 | 2 | 0 | 1 | 1 | 3 | 2 | 3 | 2 | 4 | 2 | 3 | 2 | 3 | 4 |
| **Maritime Evergreen Needleleaf Forest** | 5 | 5 | 5 | 5 | 2 | 4 | 1 | 0 | 1 | 3 | 2 | 2 | 2 | 4 | 2 | 3 | 4 | 5 | 5 |
| **Temperate Evergreen Needleleaf Forest** | 5 | 5 | 5 | 5 | 2 | 3 | 1 | 1 | 0 | 2 | 1 | 1 | 1 | 2 | 2 | 2 | 3 | 4 | 5 |
| **Temperate Deciduous Broadleaf Forest** | 5 | 5 | 5 | 5 | 4 | 3 | 3 | 3 | 2 | 0 | 1 | 1 | 2 | 1 | 2 | 2 | 3 | 4 | 5 |
| **Temperate Cool Mixed Forest** | 5 | 5 | 5 | 5 | 3 | 2 | 2 | 2 | 1 | 1 | 0 | 1 | 2 | 2 | 1 | 2 | 3 | 4 | 5 |
| **Temperate Warm Mixed Forest** | 5 | 5 | 5 | 5 | 4 | 3 | 3 | 2 | 1 | 1 | 1 | 0 | 2 | 2 | 2 | 1 | 3 | 4 | 5 |
| **Temperate Evergreen Needleleaf Woodland** | 5 | 4 | 5 | 5 | 2 | 2 | 2 | 2 | 1 | 2 | 2 | 2 | 0 | 2 | 1 | 1 | 1 | 2 | 3 |
| **Temperate Deciduous Broadleaf Woodland** | 5 | 4 | 5 | 5 | 4 | 2 | 4 | 4 | 2 | 1 | 2 | 2 | 2 | 0 | 1 | 1 | 1 | 2 | 3 |
| **Temperate Cool Mixed Woodland** | 5 | 4 | 5 | 5 | 3 | 1 | 2 | 2 | 2 | 2 | 1 | 2 | 1 | 1 | 0 | 1 | 1 | 2 | 3 |
| **Temperate Warm Mixed Woodland** | 5 | 4 | 5 | 5 | 5 | 2 | 3 | 3 | 2 | 2 | 2 | 1 | 1 | 1 | 1 | 0 | 1 | 2 | 3 |
| **Temperate Shrubland** | 5 | 3 | 5 | 5 | 5 | 3 | 2 | 4 | 3 | 3 | 3 | 3 | 1 | 1 | 1 | 1 | 0 | 1 | 2 |
| **Temperate Grassland** | 5 | 2 | 5 | 5 | 5 | 4 | 3 | 5 | 4 | 4 | 4 | 4 | 2 | 2 | 2 | 2 | 1 | 0 | 1 |
| **Temperate Desert** | 5 | 1 | 5 | 5 | 5 | 5 | 4 | 5 | 5 | 5 | 5 | 5 | 3 | 3 | 3 | 3 | 2 | 1 | 0 |
| **Subtropical Evergreen Needleleaf Forest** | 5 | 5 | 5 | 5 | 5 | 5 | 2 | 2 | 1 | 3 | 2 | 2 | 2 | 4 | 3 | 3 | 3 | 4 | 5 |
| **Subtropical Deciduous Broadleaf Forest** | 5 | 5 | 5 | 5 | 5 | 5 | 4 | 4 | 3 | 2 | 2 | 2 | 3 | 2 | 3 | 3 | 3 | 4 | 5 |
| **Subtropical Evergreen Broadleaf Forest** | 5 | 5 | 5 | 5 | 5 | 5 | 4 | 4 | 3 | 3 | 3 | 3 | 4 | 4 | 3 | 3 | 3 | 4 | 5 |
| **Subtropical Mixed Forest** | 5 | 5 | 5 | 5 | 5 | 5 | 5 | 3 | 2 | 2 | 1 | 1 | 3 | 3 | 2 | 2 | 3 | 4 | 5 |
| **Subtropical Evergreen Needleleaf Woodland** | 5 | 5 | 5 | 5 | 5 | 5 | 3 | 2 | 2 | 3 | 3 | 3 | 1 | 3 | 2 | 2 | 2 | 3 | 4 |
| **Subtropical Deciduous Broadleaf Woodland** | 5 | 4 | 5 | 5 | 5 | 5 | 5 | 4 | 4 | 1 | 3 | 3 | 3 | 1 | 2 | 2 | 2 | 3 | 4 |
| **Subtropical Evergreen Broadleaf Woodland** | 5 | 4 | 5 | 5 | 5 | 5 | 5 | 4 | 4 | 2 | 4 | 4 | 4 | 3 | 3 | 3 | 2 | 3 | 4 |
| **Subtropical Mixed Woodland** | 5 | 4 | 5 | 5 | 5 | 5 | 4 | 3 | 3 | 3 | 2 | 2 | 2 | 2 | 1 | 1 | 2 | 3 | 4 |
| **Subtropical Shrubland** | 5 | 3 | 5 | 5 | 5 | 5 | 5 | 4 | 4 | 4 | 4 | 4 | 2 | 2 | 2 | 2 | 1 | 2 | 3 |
| **Subtropical Grassland** | 5 | 2 | 5 | 5 | 5 | 5 | 5 | 5 | 5 | 5 | 5 | 5 | 3 | 3 | 3 | 3 | 2 | 1 | 2 |
| **Subtropical Desert** | 5 | 1 | 5 | 5 | 5 | 5 | 5 | 5 | 5 | 5 | 5 | 5 | 4 | 4 | 4 | 4 | 3 | 2 | 1 |
| **Tropical Evergreen Broadleaf Forest** | 5 | 5 | 5 | 5 | 5 | 5 | 5 | 5 | 5 | 5 | 5 | 5 | 5 | 5 | 5 | 5 | 5 | 5 | 5 |
| **Tropical Deciduous Woodland** | 5 | 5 | 5 | 5 | 5 | 5 | 5 | 5 | 5 | 5 | 5 | 5 | 5 | 5 | 5 | 5 | 5 | 5 | 5 |
| **Tropical Savanna** | 5 | 3 | 5 | 5 | 5 | 5 | 5 | 5 | 5 | 5 | 5 | 5 | 5 | 5 | 5 | 5 | 5 | 5 | 5 |
| **Tropical Shrubland** | 5 | 3 | 5 | 5 | 5 | 5 | 5 | 5 | 5 | 5 | 5 | 5 | 5 | 5 | 5 | 5 | 5 | 5 | 5 |
| **Tropical Grassland** | 5 | 2 | 5 | 5 | 5 | 5 | 5 | 5 | 5 | 5 | 5 | 5 | 5 | 5 | 5 | 5 | 5 | 5 | 5 |
| **Tropical Desert** | 5 | 1 | 5 | 5 | 5 | 5 | 5 | 5 | 5 | 5 | 5 | 5 | 5 | 5 | 5 | 5 | 5 | 5 | 5 |
| **Cool Needleleaf Forest** | 5 | 5 | 5 | 5 | 2 | 3 | 4 | 1 | 1 | 3 | 1 | 2 | 2 | 4 | 3 | 4 | 5 | 5 | 5 |
| **Agriculture and Grazing** | 5 | 5 | 5 | 5 | 5 | 5 | 5 | 5 | 5 | 5 | 5 | 5 | 5 | 5 | 5 | 5 | 5 | 5 | 5 |
|  | **Subtropical Evergreen Needleleaf Forest** | **Subtropical Deciduous Broadleaf Forest** | **Subtropical Evergreen Broadleaf Forest** | **Subtropical Mixed Forest** | **Subtropical Evergreen Needleleaf Woodland** | **Subtropical Deciduous Broadleaf Woodland** | **Subtropical Evergreen Broadleaf Woodland** | **Subtropical Mixed Woodland** | **Subtropical Shrubland** | **Subtropical Grassland** | **Subtropical Desert** | **Tropical Evergreen Broadleaf Forest** | **Tropical Deciduous Woodland** | **Tropical Savanna** | **Tropical Shrubland** | **Tropical Grassland** | **Tropical Desert** | **Cool Needleleaf Forest** | **Agriculture and Grazing** |
| **Undefined** | 5 | 5 | 5 | 5 | 5 | 5 | 5 | 5 | 5 | 5 | 5 | 5 | 5 | 5 | 5 | 5 | 5 | 5 | 5 |
| **Barren** | 5 | 5 | 5 | 5 | 5 | 4 | 4 | 4 | 3 | 2 | 1 | 5 | 5 | 3 | 3 | 2 | 1 | 5 | 5 |
| **Tundra** | 5 | 5 | 5 | 5 | 5 | 5 | 5 | 5 | 5 | 5 | 5 | 5 | 5 | 5 | 5 | 5 | 5 | 5 | 5 |
| **Taiga Tundra** | 5 | 5 | 5 | 5 | 5 | 5 | 5 | 5 | 5 | 5 | 5 | 5 | 5 | 5 | 5 | 5 | 5 | 5 | 5 |
| **Boreal Evergreen Needleleaf Forest** | 5 | 5 | 5 | 5 | 5 | 5 | 5 | 5 | 5 | 5 | 5 | 5 | 5 | 5 | 5 | 5 | 5 | 2 | 5 |
| **Boreal Mixed Woodland** | 5 | 5 | 5 | 5 | 5 | 5 | 5 | 5 | 5 | 5 | 5 | 5 | 5 | 5 | 5 | 5 | 5 | 3 | 5 |
| **Subalpine** | 2 | 4 | 4 | 5 | 3 | 5 | 5 | 4 | 5 | 5 | 5 | 5 | 5 | 5 | 5 | 5 | 5 | 4 | 5 |
| **Maritime Evergreen Needleleaf Forest** | 2 | 4 | 4 | 3 | 2 | 4 | 4 | 3 | 4 | 5 | 5 | 5 | 5 | 5 | 5 | 5 | 5 | 1 | 5 |
| **Temperate Evergreen Needleleaf Forest** | 1 | 3 | 3 | 2 | 2 | 4 | 4 | 3 | 4 | 5 | 5 | 5 | 5 | 5 | 5 | 5 | 5 | 1 | 5 |
| **Temperate Deciduous Broadleaf Forest** | 3 | 2 | 3 | 2 | 3 | 1 | 2 | 3 | 4 | 5 | 5 | 5 | 5 | 5 | 5 | 5 | 5 | 3 | 5 |
| **Temperate Cool Mixed Forest** | 2 | 2 | 3 | 1 | 3 | 3 | 4 | 2 | 4 | 5 | 5 | 5 | 5 | 5 | 5 | 5 | 5 | 1 | 5 |
| **Temperate Warm Mixed Forest** | 2 | 2 | 3 | 1 | 3 | 3 | 4 | 2 | 4 | 5 | 5 | 5 | 5 | 5 | 5 | 5 | 5 | 2 | 5 |
| **Temperate Evergreen Needleleaf Woodland** | 2 | 3 | 4 | 3 | 1 | 3 | 4 | 2 | 2 | 3 | 4 | 5 | 5 | 5 | 5 | 5 | 5 | 2 | 5 |
| **Temperate Deciduous Broadleaf Woodland** | 4 | 2 | 4 | 3 | 3 | 1 | 3 | 2 | 2 | 3 | 4 | 5 | 5 | 5 | 5 | 5 | 5 | 4 | 5 |
| **Temperate Cool Mixed Woodland** | 3 | 3 | 3 | 2 | 2 | 2 | 3 | 1 | 2 | 3 | 4 | 5 | 5 | 5 | 5 | 5 | 5 | 3 | 5 |
| **Temperate Warm Mixed Woodland** | 3 | 3 | 3 | 2 | 2 | 2 | 3 | 1 | 2 | 3 | 4 | 5 | 5 | 5 | 5 | 5 | 5 | 4 | 5 |
| **Temperate Shrubland** | 3 | 3 | 3 | 3 | 2 | 2 | 2 | 2 | 1 | 2 | 3 | 5 | 5 | 5 | 5 | 5 | 5 | 5 | 5 |
| **Temperate Grassland** | 4 | 4 | 4 | 4 | 3 | 3 | 3 | 3 | 2 | 1 | 2 | 5 | 5 | 5 | 5 | 5 | 5 | 5 | 5 |
| **Temperate Desert** | 5 | 5 | 5 | 5 | 4 | 4 | 4 | 4 | 3 | 2 | 1 | 5 | 5 | 5 | 5 | 5 | 5 | 5 | 5 |
| **Subtropical Evergreen Needleleaf Forest** | 0 | 2 | 2 | 1 | 1 | 3 | 3 | 2 | 3 | 4 | 5 | 2 | 3 | 4 | 4 | 5 | 5 | 1 | 5 |
| **Subtropical Deciduous Broadleaf Forest** | 2 | 0 | 1 | 1 | 3 | 2 | 3 | 2 | 3 | 4 | 5 | 2 | 2 | 3 | 4 | 5 | 5 | 3 | 5 |
| **Subtropical Evergreen Broadleaf Forest** | 2 | 1 | 0 | 1 | 2 | 2 | 1 | 2 | 3 | 4 | 5 | 1 | 3 | 3 | 4 | 5 | 5 | 3 | 5 |
| **Subtropical Mixed Forest** | 1 | 1 | 1 | 0 | 2 | 2 | 2 | 1 | 3 | 4 | 5 | 2 | 3 | 4 | 4 | 5 | 5 | 5 | 5 |
| **Subtropical Evergreen Needleleaf Woodland** | 1 | 3 | 2 | 2 | 0 | 2 | 2 | 1 | 1 | 2 | 3 | 3 | 4 | 5 | 5 | 5 | 5 | 4 | 5 |
| **Subtropical Deciduous Broadleaf Woodland** | 3 | 2 | 2 | 2 | 2 | 0 | 1 | 2 | 1 | 2 | 3 | 2 | 3 | 4 | 4 | 5 | 5 | 2 | 5 |
| **Subtropical Evergreen Broadleaf Woodland** | 3 | 3 | 1 | 2 | 2 | 1 | 0 | 1 | 2 | 3 | 4 | 2 | 1 | 2 | 2 | 3 | 4 | 4 | 5 |
| **Subtropical Mixed Woodland** | 2 | 2 | 2 | 1 | 1 | 2 | 1 | 0 | 1 | 2 | 3 | 3 | 2 | 2 | 2 | 3 | 4 | 4 | 5 |
| **Subtropical Shrubland** | 3 | 3 | 3 | 3 | 1 | 1 | 2 | 1 | 0 | 1 | 2 | 4 | 3 | 2 | 1 | 2 | 3 | 5 | 5 |
| **Subtropical Grassland** | 4 | 4 | 4 | 4 | 2 | 2 | 3 | 2 | 1 | 0 | 1 | 4 | 3 | 2 | 2 | 1 | 2 | 5 | 5 |
| **Subtropical Desert** | 5 | 5 | 5 | 5 | 3 | 3 | 4 | 3 | 2 | 1 | 0 | 5 | 4 | 3 | 3 | 2 | 1 | 5 | 5 |
| **Tropical Evergreen Broadleaf Forest** | 2 | 2 | 1 | 2 | 3 | 2 | 2 | 3 | 4 | 4 | 5 | 0 | 1 | 2 | 2 | 3 | 4 | 5 | 5 |
| **Tropical Deciduous Woodland** | 3 | 2 | 3 | 3 | 4 | 3 | 1 | 2 | 3 | 3 | 4 | 1 | 0 | 1 | 1 | 2 | 3 | 5 | 5 |
| **Tropical Savanna** | 4 | 3 | 3 | 4 | 5 | 4 | 2 | 2 | 2 | 2 | 3 | 2 | 1 | 0 | 1 | 1 | 2 | 5 | 5 |
| **Tropical Shrubland** | 4 | 4 | 4 | 4 | 5 | 4 | 2 | 2 | 1 | 2 | 3 | 2 | 1 | 1 | 0 | 1 | 2 | 5 | 5 |
| **Tropical Grassland** | 5 | 5 | 5 | 5 | 5 | 5 | 3 | 3 | 2 | 1 | 2 | 3 | 2 | 1 | 1 | 0 | 1 | 5 | 5 |
| **Tropical Desert** | 5 | 5 | 5 | 5 | 5 | 5 | 4 | 4 | 3 | 2 | 1 | 4 | 3 | 2 | 2 | 1 | 0 | 5 | 5 |
| **Cool Needleleaf Forest** | 1 | 3 | 3 | 5 | 4 | 2 | 4 | 4 | 5 | 5 | 5 | 5 | 5 | 5 | 5 | 5 | 5 | 0 | 5 |
| **Agriculture and Grazing** | 5 | 5 | 5 | 5 | 5 | 5 | 5 | 5 | 5 | 5 | 5 | 5 | 5 | 5 | 5 | 5 | 5 | 5 | 0 |
