# Supplementary figures and images for: A fuzzy logic decision support model for climate-driven biomass loss risk in western Oregon and Washington
Source: PLoS One. 2019 Oct 25;14(10):e0222051. doi: 10.1371/journal.pone.0222051 (PMC6814215; doi:10.1371/journal.pone.0222051)

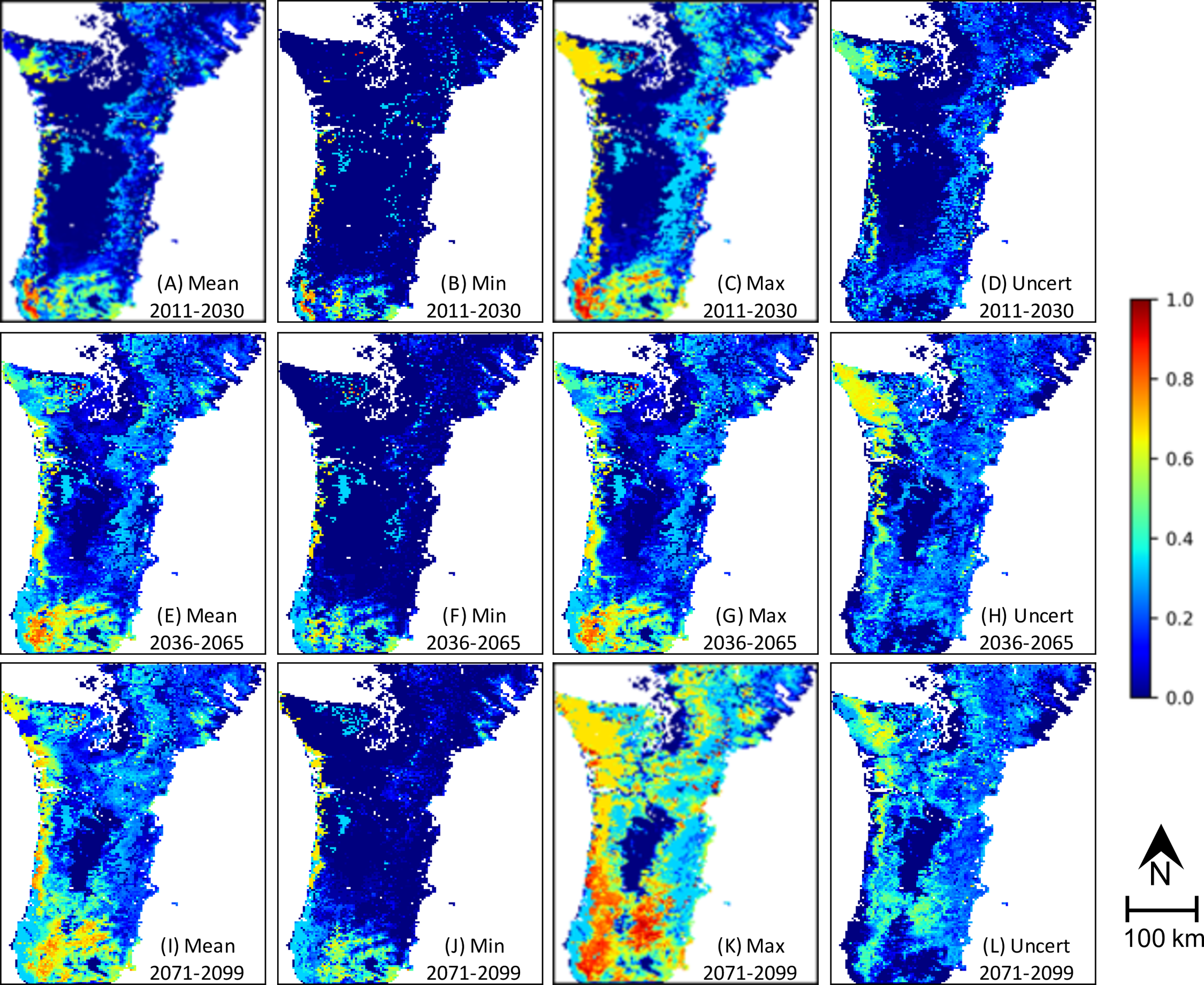

Supplement: S1 Fig — Figure rows include the mean, minimum, maximum, and uncertainty representation for one time period. (min: minimum; max: maximum; uncert: uncertainty). (TIF) [file pone.0222051.s002.tif]

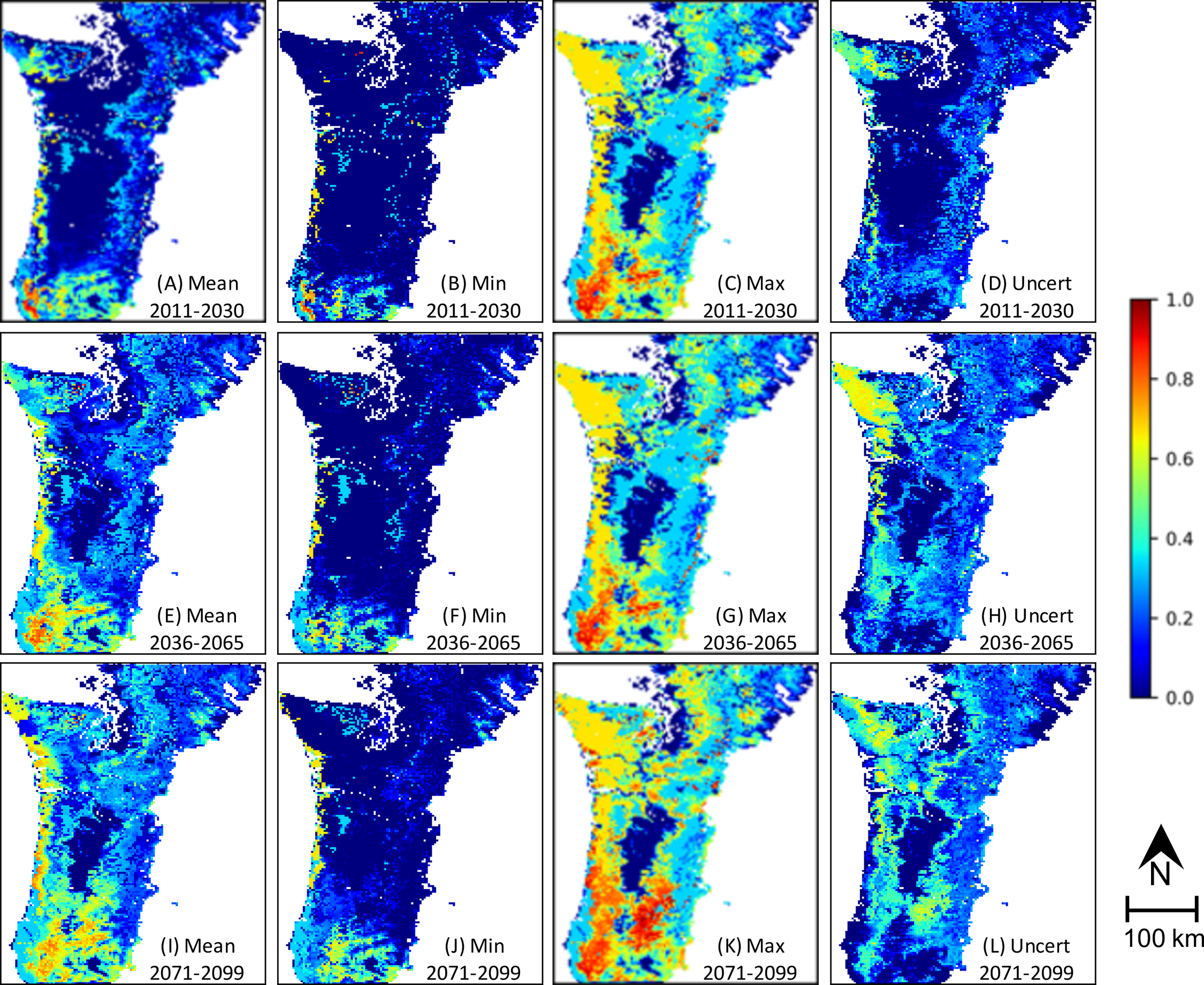

Supplement: S2 Fig — Figure rows include the mean, minimum, maximum, and uncertainty representation for one time period. (min: minimum; max: maximum; uncert: uncertainty). (TIF) [file pone.0222051.s003.tif]

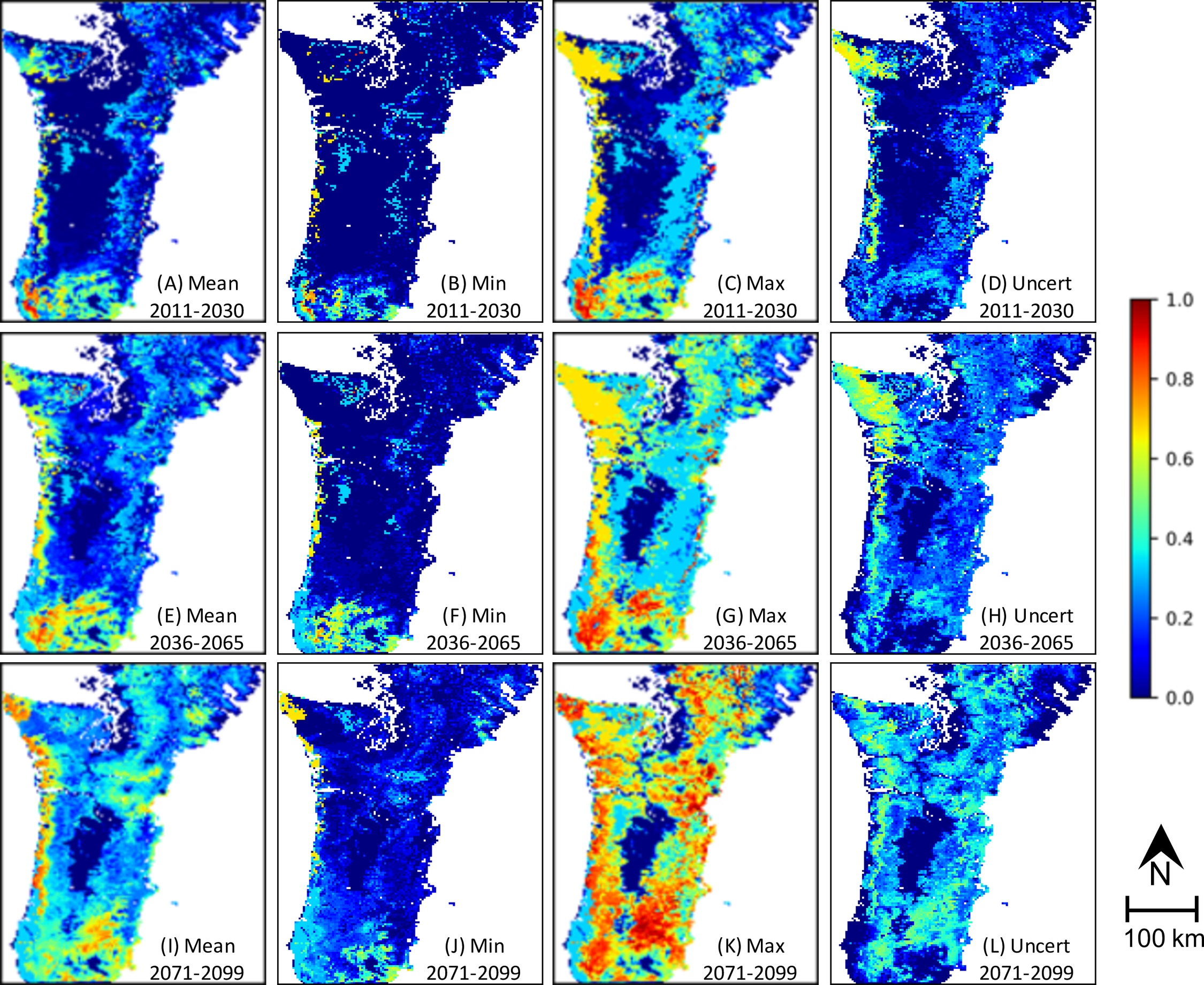

Supplement: S3 Fig — Figure rows include the mean, minimum, maximum, and uncertainty representation for one time period. (min: minimum; max: maximum; uncert: uncertainty). (TIF) [file pone.0222051.s004.tif]

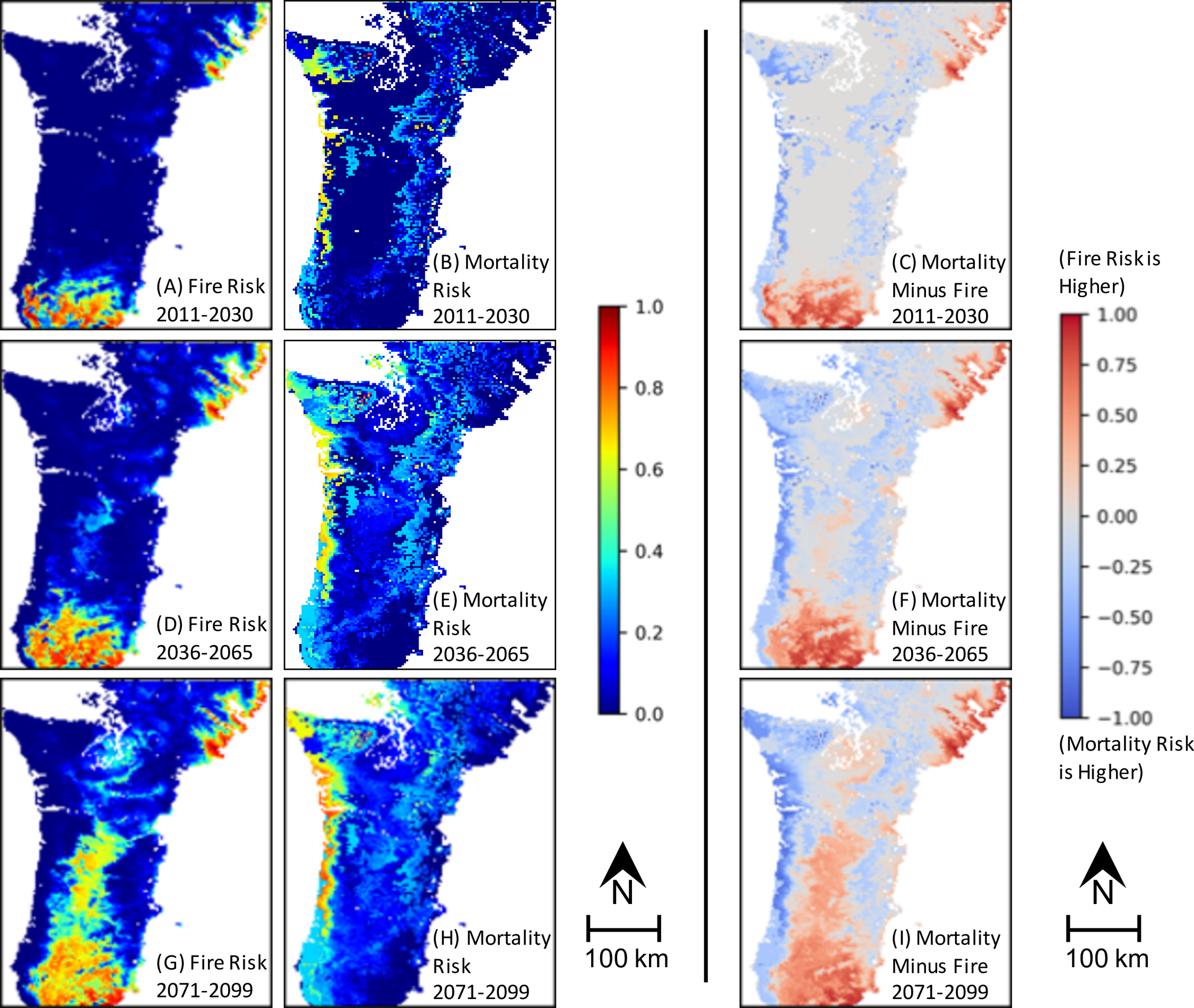

Supplement: S4 Fig — Maps of MC2 Fire Loss Risk (A, D, G), MC2 Mortality Risk (B, E, H), and MC2 Fire Loss Risk minus MC2 Mortality Risk (C, F, I) from EEMS model for the RCP 4.5 FS scenario. Figure rows represent time periods. (TIF) [file pone.0222051.s005.tif]

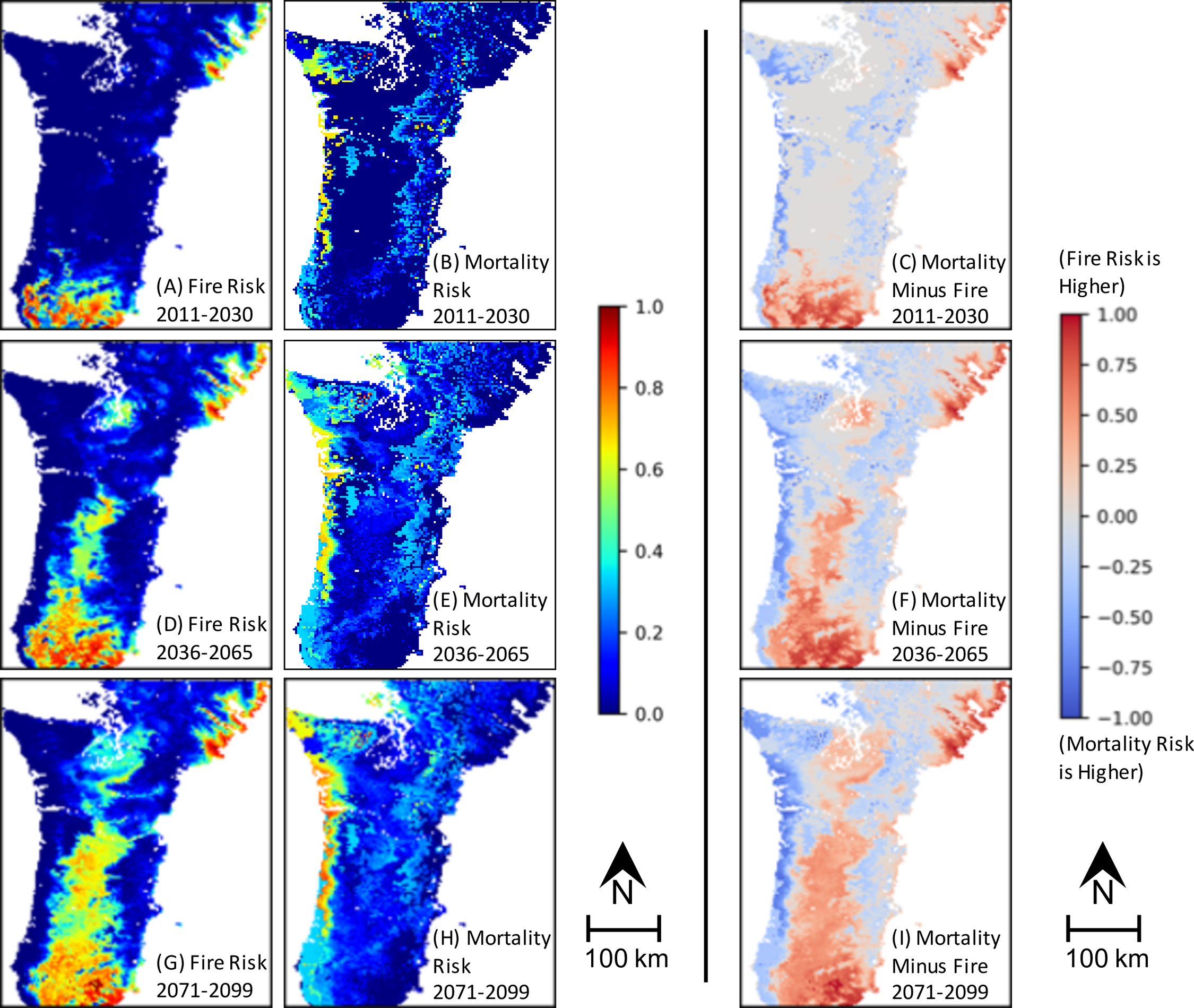

Supplement: S5 Fig — Maps of MC2 Fire Loss Risk (A, D, G), MC2 Mortality Risk (B, E, H), and MC2 Fire Loss Risk minus MC2 Mortality Risk (C, F, I) from EEMS model for the RCP 4.5 NFS scenario. Figure rows represent time periods. (TIF) [file pone.0222051.s006.tif]

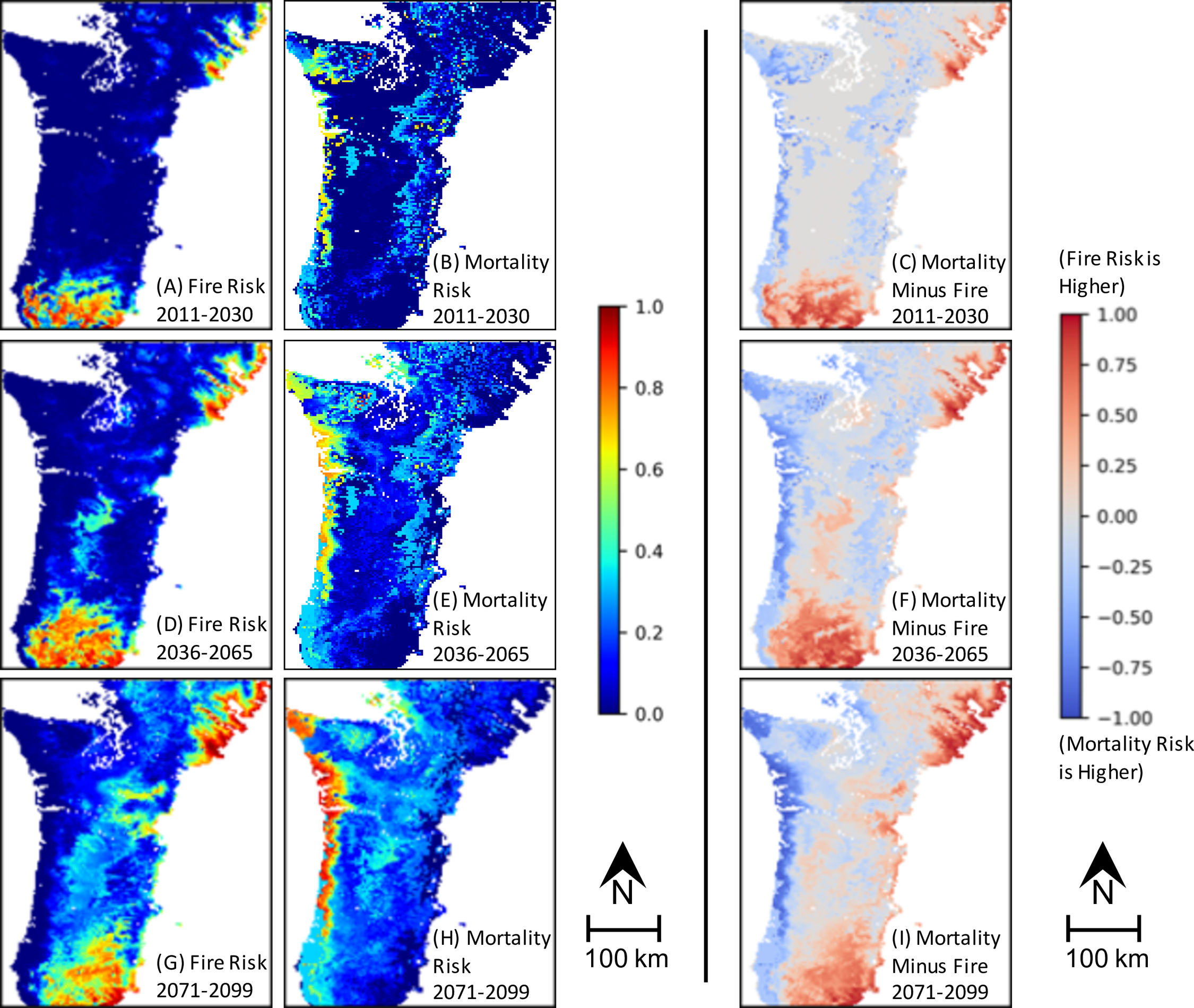

Supplement: S6 Fig — Maps of MC2 Fire Loss Risk (A, D, G), MC2 Mortality Risk (B, E, H), and MC2 Fire Loss Risk minus MC2 Mortality Risk (C, F, I) from EEMS model for the RCP 8.5 FS scenario. Figure rows represent time periods. (TIF) [file pone.0222051.s007.tif]

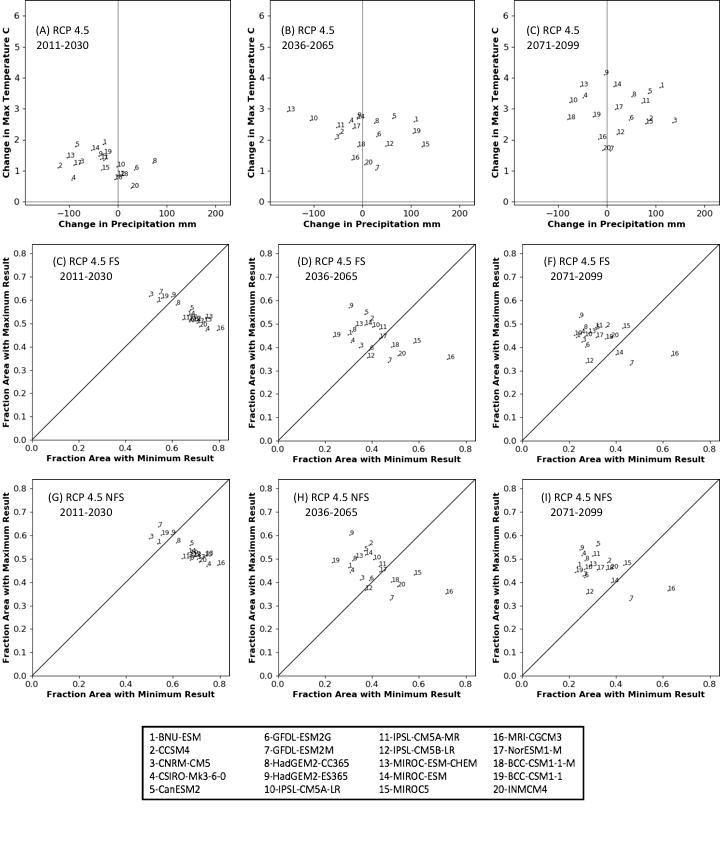

Supplement: S7 Fig — Change (1971–2000 vs future period) in average maximum temperature vs change in average annual precipitation for each of the 20 RCP 4.5 climate futures (A-C), and fraction of the simulated area with maximum and minimum values MC2 Biomass Loss Risk for the RCP 4.5 FS scenario (D-F) and the NFS scenario (G-I). In graphs D-I, a point above the 45° line indicates that the results of the MC2 run driven by that climate future showed a greater number of high vs low values of biomass loss over more of the study area. Points below the 45° line indicate that MC2 results showed a greater number of low vs high values over more of the area. (mm: millimeters). (TIFF) [file pone.0222051.s008.tiff]
